# Essential ion binding residues for Na^+^ flow in stator complex of the *Vibrio* flagellar motor

**DOI:** 10.1038/s41598-019-46038-6

**Published:** 2019-08-02

**Authors:** Yasuhiro Onoue, Masayo Iwaki, Ai Shinobu, Yasutaka Nishihara, Hiroto Iwatsuki, Hiroyuki Terashima, Akio Kitao, Hideki Kandori, Michio Homma

**Affiliations:** 1 Division of Biological Science, Graduate School of Science, Nagoya University, Furo-cho, Chikusa-ku, Nagoya 464-8602 Japan; 2 Department of Life Science and Applied Chemistry, Nagoya Institute of Technology, Showa-ku, Nagoya 466-8555 Japan; 3 Institute of Molecular and Cellular Biosciences, The University of Tokyo, 1-1-1 Yayoi, Bunkyo, Tokyo 113-0032 Japan; 4 School of Life Science and Technology, Tokyo Institute of Technology, 2-12-1 Ookayama, Meguro, Tokyo 152-8550 Japan

**Keywords:** Bioenergetics, Bacterial structural biology

## Abstract

The bacterial flagellar motor is a unique supramolecular complex which converts ion flow into rotational force. Many biological devices mainly use two types of ions, proton and sodium ion. This is probably because of the fact that life originated in seawater, which is rich in protons and sodium ions. The polar flagellar motor in *Vibrio* is coupled with sodium ion and the energy converting unit of the motor is composed of two membrane proteins, PomA and PomB. It has been shown that the ion binding residue essential for ion transduction is the conserved aspartic acid residue (PomB-D24) in the PomB transmembrane region. To reveal the mechanism of ion selectivity, we identified essential residues, PomA-T158 and PomA-T186, other than PomB-D24, in the Na^+^-driven flagellar motor. It has been shown that the side chain of threonine contacts Na^+^ in Na^+^-coupled transporters. We monitored the Na^+^-binding specific structural changes using ATR-FTIR spectroscopy. The signals were abolished in PomA-T158A and -T186A, as well as in PomB-D24N. Molecular dynamics simulations further confirmed the strong binding of Na^+^ to D24 and showed that T158A and T186A hindered the Na^+^ binding and transportation. The data indicate that two threonine residues (PomA-T158 and PomA-T186), together with PomB-D24, are important for Na^+^ conduction in the *Vibrio* flagellar motor. The results contribute to clarify the mechanism of ion recognition and conversion of ion flow into mechanical force.

## Introduction

A fundamental part of the development of life on Earth was the evolution of cells possessing a membrane between the interior and exterior environments. The exchange of substances between the interior of a cell and the external environment is essential for cell survival. The transport of substances against the concentration difference between the two regions requires energy coupling. Cells often use the driving force of ions (typically protons and sodium ions) as the energy for the transport events^1,2^. This is probably due to the fact that life originated in seawater, which is rich in protons and sodium ions. Many biological devices mainly use these two types of ions. The mechanism of the ion motive force has been best analyzed in ATPase and in the bacterial flagellar motor^3,4^. Their operating mechanisms and structures are totally different, but both are rotary motors that use proton and sodium ions as the main coupling ions to generate the rotary force.

The bacterial flagellar motor is a supramolecular complex that rotates their filaments for bacterial swimming^5–7^. This rotary motor is composed of a stator and rotor. The rotor is located beneath the membrane-embedded flagellar basal body and faced to the cytoplasm. FliG, a main component of the rotor, participates directly in torque generation. The stator, a membrane protein complex, assembles around the rotor to interact with each other and provides a transmembrane ion-conducting pathway.

Most of the flagellar motors are powered by H^+^ or Na^+^ currents. It was recently reported that the flow of calcium ions (Ca^2+^) or magnesium ions (Mg^2+^), as well as potassium ions (K^+^) can also drive the motor^8,9^. *Escherichia coli* and *Salmonella* have H^+^-driven motors, *Vibrio alginolyticus* and alkalophilic *Bacillus* have Na^+^-driven motors, the alkaliphilic species *B. alcalophilus* has a motor coupled with K^+^ and rubidium ion (Rb^+^), and *Paenibacillus* has a motor driven by divalent cations (Ca^2+^ and Mg^2+^). The most important part of the motor required for the conversion of the ion flux mechanical energy is the stator. The stator is composed of two membrane proteins, subunit A and B that form a hetero-hexameric complex in a 4:2 ratio^10,11^. The most well studied motors are from *E. coli* and *V. alginolyticus*, which can rotate at up to 300 Hz and 1,700 Hz, respectively. The rotation of the flagellar motor is originated by conformational changes of the stator complex; these changes are coupled with the ion flow.

**Figure 1:**
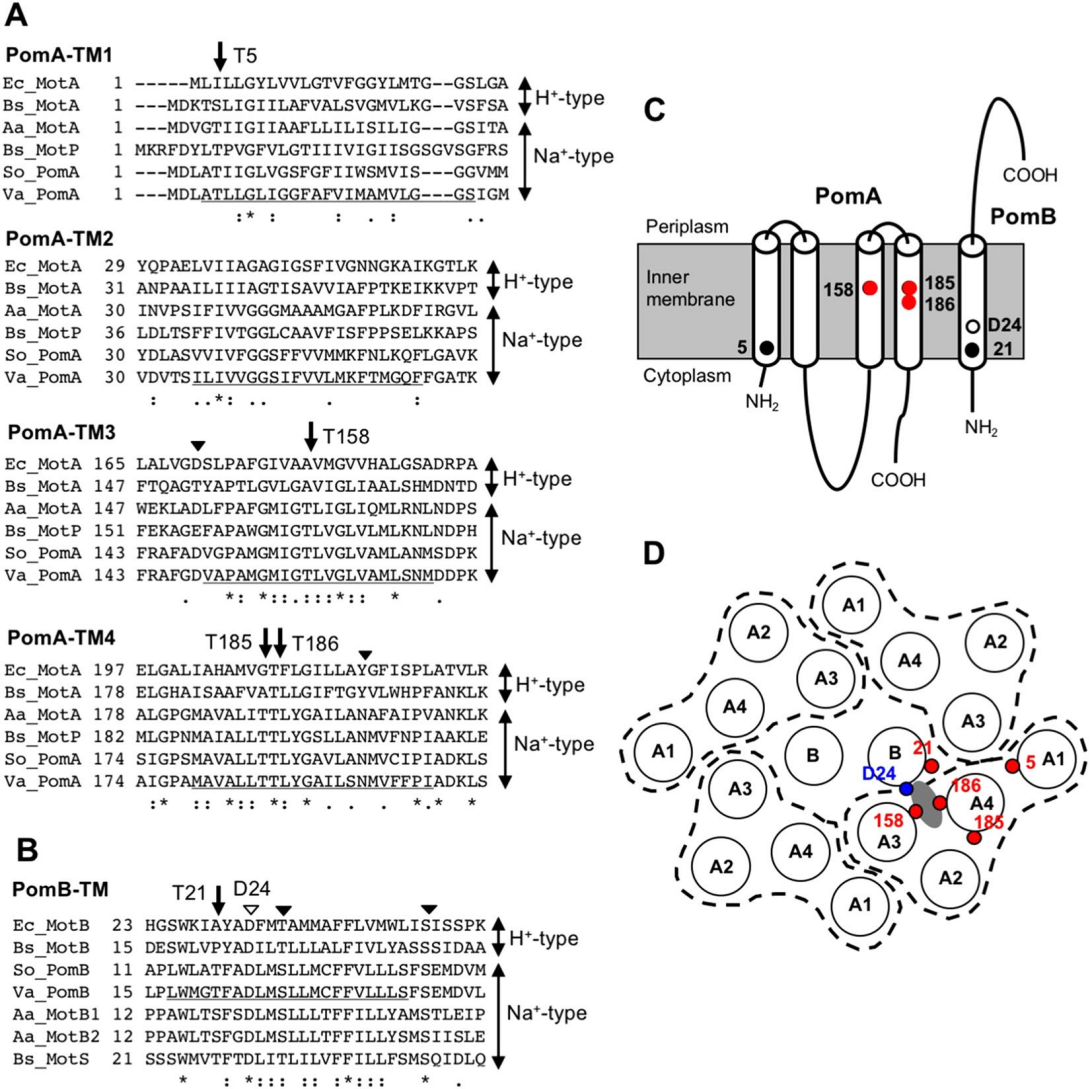
Primary and schematic structures of transmembrane regions in the stator complex of the flagellar motor. (**A**) Amino acid sequence alignment near four transmembrane regions of subunit A in the H^+^-driven and Na^+^-driven flagellar motor. Arrows indicate the conserved threonine residues in the Na^+^-type stator investigated in this study. Solid arrowheads indicate highly conserved amino acid residues that can coordinate Na^+^ and investigated previously. The underline in the *Va* sequences is the predicted transmembrane region. *Va*, *V. alginolyticus*; *So*, *S. oneidensis*; *Bs*, *B. subtilis*; *Aa*, *A. aeolicus*; *Ec*, *E. coli*. (**B**) Amino acid sequence alignment of a transmembrane region of subunit B in the H^+^-driven and Na^+^-driven flagellar motor. The arrow indicates the highly conserved threonine residue in the Na^+^-type stator investigated in this study. Solid arrowheads indicate conserved residues that can be candidate Na^+^-binding sites, and have been investigated previously. The open arrowhead is the conserved aspartic acid (D24). The underline in *Va* sequences is the predicted transmembrane region of PomB. (**C**) Schematic representation of the stator complex of Na^+^-driven flagellar motor and location of conserved threonine residues investigated in this study. (**D**) Model for the arrangement of transmembrane regions in the stator complex and position of conserved threonine residues. Model for *E. coli* MotA/MotB based on cross-linking experiments are adapted to that for PomA/PomB according to the amino acid alignment. Each circle indicates a transmembrane alpha helix and designated as A or B. PomA composed of four transmembrane regions are surrounded by the dashed line. The predicted Na^+^-binding site is gray. Red circles denote threonine residue investigated in this report. The blue circle denotes the position of the conserved aspartic acid (D24).

Subunit A has four transmembrane (TM) helixes and a large cytoplasmic domain between the second (TM-A2) and third TM (TM-A3) helices^6,11^. Subunit B has a single TM (TM-B) helix at the N-terminus, with most of the remaining structure present in the periplasmic space with a peptidoglycan binding motif. To generate the driving force of rotation, the cytoplasmic loop of subunit A interacts with the rotor protein FliG. In bacterial flagellar motors, the ion binding site in the stator complex is an aspartic acid (Asp) residue of the B subunit (D24 for *Vibrio* PomB or *E. coli* D32 for MotB: Fig. 1), although the binding of ions to the Asp residue has not been directly demonstrated^12,13^. Mutations other than conversion of the Asp residue into glutamic acid (Glu) are completely dysfunctional. Although the pathway of the coupling ion in the stator complex is unclear, the ion channel is speculated to be composed of three TM helixes; TM-B, TM-A3, and TM-A4. Proline (Pro)173 and Pro222 residues of *E. coli* MotA near the cytoplasmic side of TM-A3 and TM-A4 regulate the conformational changes required for torque generation^14,15^. The structural model of the arrangement of TM regions in MotAB complex has been constructed based on the disulfide cross-linking analysis^16–18^. The permeation process of hydronium ions and water molecules through the channel of the MotAB complex of the proton motor was examined by a steered molecular dynamics (MD) simulation^19^. The findings suggested that the Leu46 residue of MotB acts as a gate for the permeation of hydronium ions, which in turn forms a water wire. This water wire might assist the proton transfer to Asp32 on MotB. In the model, the narrowest part of the channel is located close to the periplasm around V184(TM-A3), T209(TM-A4), L42(TM-B), and L46(TM-B) of *E. coli* (Fig. 1A,B,D). The T209(TM-A4) of *E. coli* MotA corresponds to T186(TM-A4) of *V. alginolyticus* PomA. For the sodium driven type of stator complex, the PomA-D148, PomA-N194, PomA-L183, PomB-F22, and PomB-C31 residues in the TM regions are involved in the ion translocation and have been suggested to form an ion channel^7^. It has been proposed that the PomB-S26 of *V. cholerae*, which corresponds to PomB-S27 of *V. alginolyticus*, is a crucial residue to perturb the hydration shell of Na^+^ in the stator channel^20^.

Almost all mutations of the residues in the TM regions do not yield a phenotype similar to the Asp mutation of TM-B, and motility is retained. Some exceptions are PomA-T158I and PomA-T186I that were isolated by random screening and seem to have no function on soft agar^21^. This phenotype resembles that of the PomB-D24 mutants. By solving the crystal structures of sodium ion-conducting membrane proteins, it has revealed that the sodium ion is bound to specific sites in the TM regions: oxygen atoms of the side-chain carboxyl or hydroxyl groups, and of main-chain carbonyl groups^22–25^. In this study, we focused on the threonine (Thr) residues, which are candidate residues for the ion binding in the stator complex of PomAB.

## Results

### Motility of Thr mutants

**Figure 2:**
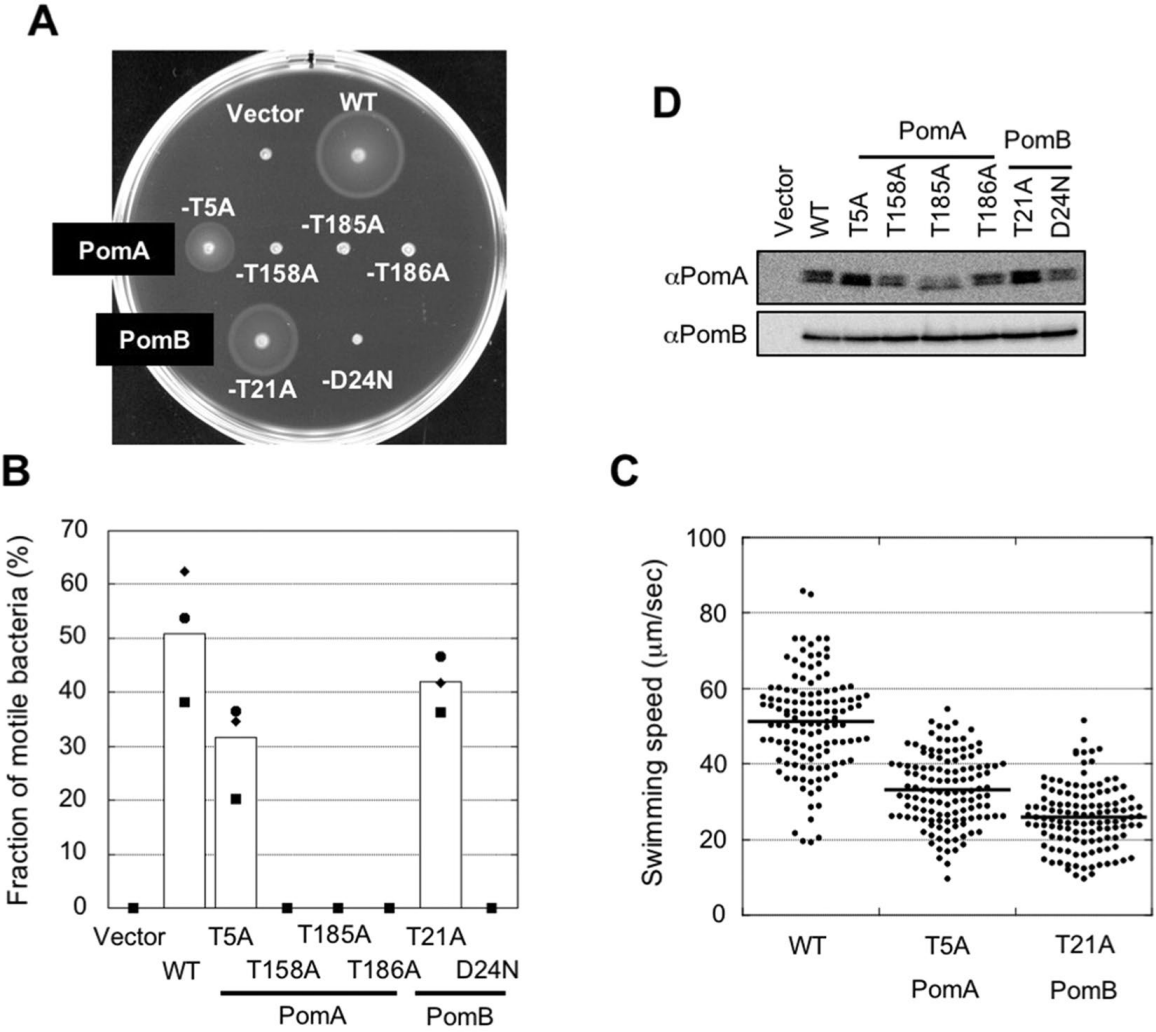
Motility of threonine mutants. (**A**) Motility on soft-agar plates of the threonine mutants. Overnight cultures were inoculated in soft-agar plates (0.25% agar) and incubated at 30 °C for 5 h. (**B**) Motility fraction of threonine mutants in the liquid medium. After 4 h of the second culture, motility was examined by light microscopy. Squares, diamonds, and circles indicate data of three independent experiments and the white bars are the total data. Numbers of bacteria examined in total are 482, 313, 390, 351, 378, 369, 291, and 350 for Vector, WT, PomA-T5A, PomA-T158A, PomA-T185A, PomA-T186A, PomB-T21A, and PomB-D24N, respectively. (**C**) Swimming speed of threonine mutants in the liquid medium. Thick horizontal lines are the average swimming speed. Number of bacteria examined in total is 125. (**D**) Expression level of stator proteins in threonine mutants. After growth for 4 h, cells were collected. The proteins were separated by SDS-PAGE and the gels were subjected to Western blotting using anti-PomA or PomB antibody. The regions of interest were cropped from the blotting images. The full-length blots are presented in Fig. S14(A).

Stator complexes are driven by the Na^+^ motive force and have four conserved Thr residues in PomA and a highly conserved Thr residue in PomB in the TM regions (Fig. 1). Furthermore, there is no other conserved polar-residues in the TM regions whose side chain could be a potential candidate to contribute the Na^+^ transport except a main-chain carbonyl. We introduced alanine (Ala) mutations into these residues to reveal their functions. The PomB-D24N mutant was used for comparison. On soft-agar plates, PomA-T5A and PomB-T21A retained motility (Fig. 2A). These mutants swam in the liquid, although the swimming speeds were less than the wild type (WT) (Fig. 2B,C). PomA-T158A, PomA-T185A, and PomA-T186A did not spread in the soft-agar plates and nor did PomB-D24N. Furthermore, these mutants lost motility completely in liquid medium (Fig. 2B). The results indicate that the Thr residues at 158, 185, and 186 on PomA are critical for the motor function.

### Ion permeability of Thr mutants

We tried to detect the ion transport activity of the Thr mutants by measuring bacterial growth. PomB has an amphipathic helix in the periplasmic region called the plug region^26^, which suppresses the ion leakage of the stator complex when the complex is dissociated from the flagellar motor. When the plug-less PomA/PomB complex is expressed in either a flagellated or a non-flagellated *E. coli* strain, bacterial growth was inhibited because of the ion leakage into the cytoplasm^27,28^.

**Figure 3:**
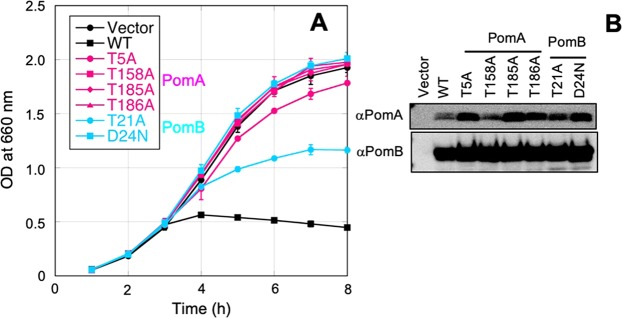
Na^+^ transport activity observed according to the growth rate of bacteria. (**A**) Growth curves of *E. coli* cells in which threonine mutants of plug-less stator complex are expressed. Overnight cultures were diluted into fresh culture with 0.2% arabinose at an OD_660_ of 0.02 and shaken at 37 °C. The OD_660_ was measured every hour. Average and standard deviation of three independent experiments are plotted. Mutants on PomA and PomB are shown in magenta and cyan plots, respectively. (**B**) Expression level of threonine mutants of plug-less stator complex. After grown for 4 h, cells were collected. The proteins were separated by SDS-PAGE and the gels were subjected to Western blotting using anti-PomA or PomB antibody. The regions of interest were cropped from the blotting images. The full-length blots are presented in Fig. S14(B).

We expressed the plug-less PomA/PomB with Thr mutants in *E. coli* strain DH5α and obtained bacterial growth curves (Fig. 3). The PomA-T158A, PomA-T185A, and PomA-T186A mutants grew well similar to the vector control and the PomB-D24N mutant, suggesting the near-complete loss of the ion transport activity by the mutations. Growth of the PomA-T5A and PomB-T21A mutants was impeded, but not to the same extent as the wild type. This may suggest that the motility defect in these mutants is caused by defective ion transport activity.

### Stable stator complex formation of Thr mutants

**Figure 4:**
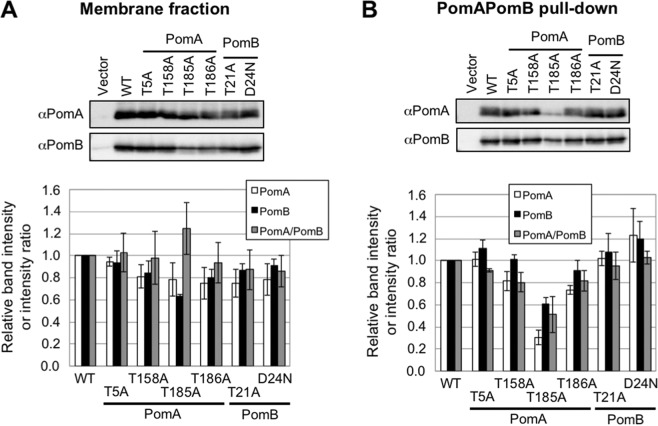
Complex formation between PomA and PomB. (**A**) The membrane fraction of threonine mutants in *E. coli* was analyzed by Western blotting using anti-PomA and -PomB antibodies (top). The regions of interest were cropped from the blotting images. The band intensity and intensity ratio are presented (bottom). The full-length blots are presented in Fig. S15(A). White, black, and gray bars are intensities of PomA and PomB, and their ratio (PomA/PomB), respectively. Each value is the average of three independent experiments and the standard deviations are shown in vertical bars. (**B**) After the membrane fractions of threonine mutants were solubilized with DMNG, PomB was pulled-down using the his-tag. Western blotting using anti-PomA and -PomB antibodies which regions of interest were cropped from the blotting images (top) and band intensity and intensity ratio (bottom) are presented. Full-length blots are presented in Fig. S15(B). White, black, and gray bars are intensity of PomA and PomB, and their ratio (PomA/PomB), respectively.

The stator complex forms a stable heterohexamer, even in detergent micelles^10,29^. We examined whether the Thr mutants similarly form a stable complex. We fused histidine tag (His6) to the C-terminus of PomB and expressed the Thr mutants in *E. coli* under the control of a cold-shock promoter. The abundance of all the Thr mutants in the membrane fraction was almost identical (Fig. 4A), suggesting that the expression, membrane localization, and stability are almost not affected by mutations. Next, we solubilized the stator complex with dodecyl maltose neopentyl glycol (DMNG), purified using Ni-nitrilotriacetic acid (NTA) beads, and analyzed by immuno-blotting (Fig. 4B). In the PomA-T185 A mutant, PomA was not co-purified as in the WT. These results suggest that PomA-T185 may contribute to the formation of a stable complex.

### Attenuated total reflectance-Fourier transform infrared (ATR-FTIR) spectroscopy

The PomA-T158A and PomA-T186A mutants were completely incapable of motility and ion transport activity, but could form the stable complex even in detergent micelle, as observed with the PomB-D24N mutant. We hypothesized that these residues are involved in Na^+^-binding. To test this hypothesis, we examined the Na^+^-binding capability of purified PomA/PomB by ATR-FTIR spectroscopy^30^. Cation binding was assessed at a physiologically relevant neutral pH (7.0) to maintain the protein structure properly, rather than at the acidic pH (5.5), at which the protein structure may not be equivalent to that in the natural condition, used in the previous experiments. The quality of protein purification was also largely improved using the new plasmid construct and the detergent (Fig. S1).

**Figure 5:**
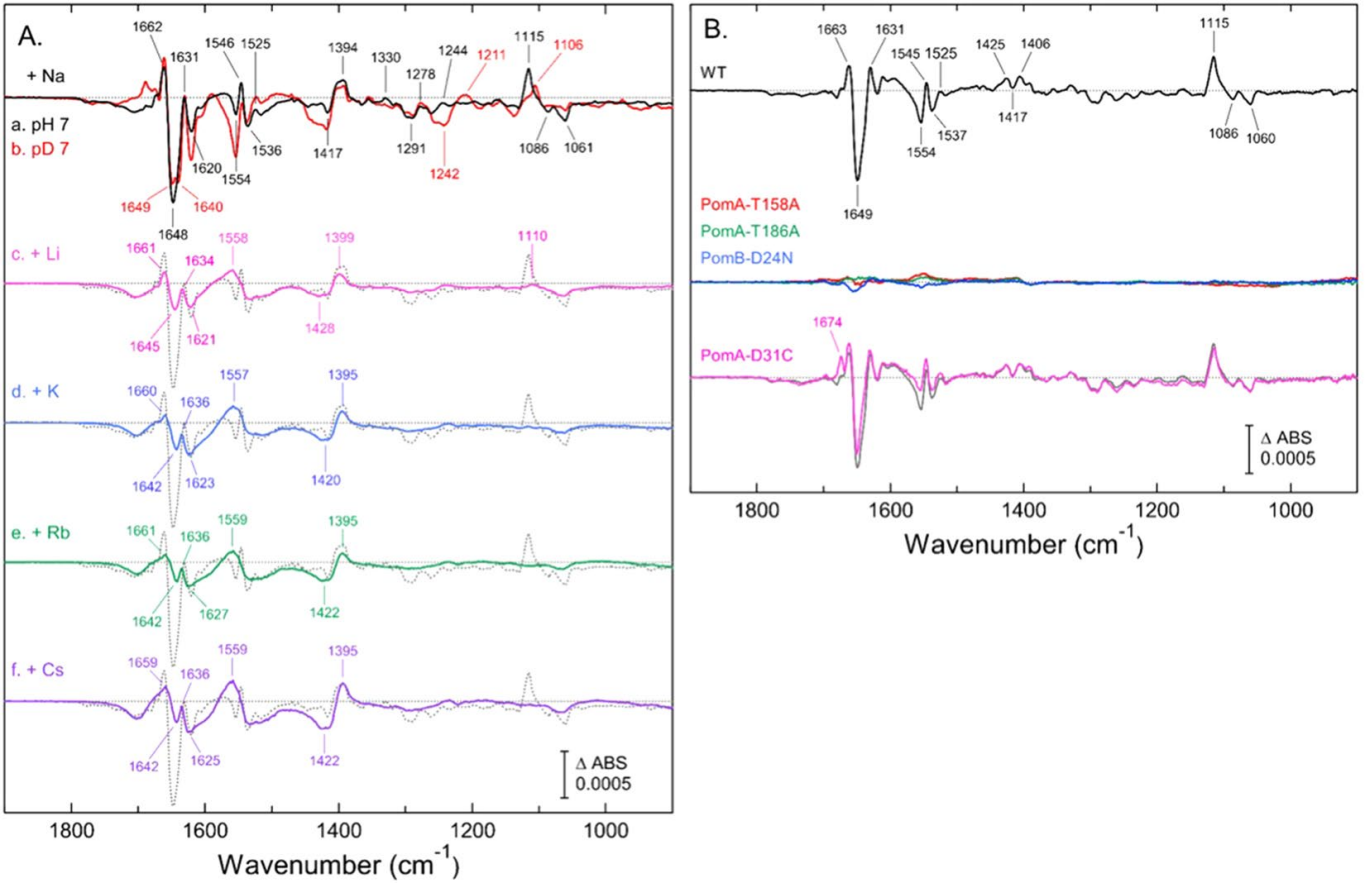
Cation-induced difference ATR-FTIR spectra of PomA/PomB. In (**A**) difference ATR-FTIR spectra of “20 mM -*minus*- 0 mM” NaCl (trace-a., black), LiCl (c., magenta), KCl (d., blue), RbCl (e., green) or CsCl (f., purple) in the WT PomA/PomB were measured in H_2_O medium (10 mM MOPS-Tris, pH 7.0, 5 mM MgCl_2_). The Na^+^-induced spectrum in D_2_O medium at pD 7.0 (b., red) is overlaid to the trace-a. All the spectra had been subtracted by baseline drifts as described in the experimental section. For comparison, the Na^+^-induced spectrum (trace a) is overlaid to traces (b.)–(f.) (dotted line). In (**B**) the double differences of 20 mM “NaCl -*minus*- KCl” were calculated in the WT (black, upper panel) and in the mutants (middle and lower panels) of PomA-T158A (red), PomA-T186A (green), PomB-D24N (blue), and PomA-D31C (magenta). Each spectrum was obtained by subtraction of the spectrum of “20 mM -*minus*- 0 mM KCl” from the “20 mM -*minus*- 0 mM NaCl”. The original Na^+^- and K^+^-induced spectra of WT are the same in trace-a. and -d. In (**A**) respectively. For comparison, the trace of WT (gray) is overlaid to that of PomA-D31C. For the Na^+^- and K^+^-induced spectra of the mutants, which were used for calculation, are shown in Fig. S2.

The trace-a in Fig. 5A shows the difference of the ATR-FTIR spectrum of “20 mM -*minus*- 0 mM” NaCl in the WT PomA/PomB at pH 7.0 in a water (H_2_O) medium. Prominent IR features were observed in the amide I region at 1662(+), 1648(−), and 1631(+) cm^−1^; in the amide II and/or carboxylate antisymmetric stretch (ν_as_) region at 1554(−), 1546(+), 1536(−), and 1525(+) cm^−1^; and in the carboxylate symmetric stretch (ν_s_) region at 1417(−) and 1394(+) cm^−1^. In a lower frequency region, the bands at 1115(+), 1086(−), and 1061(−) cm^−1^ were tentatively assigned to the Thr sidechain CO stretch, according to the literature^31^. Comparison with the spectrum in deuterium (D_2_O) medium (trace-b., Fig. 5A) identified some H/D sensitive IR bands, which indicated that proton-coupled molecular structural changes were involved in the Na^+^-binding. The most intensely negative band at 1648 cm^−1^ in H_2_O seemed to split into two bands at 1649(−) and 1640(−) cm^−1^ in D_2_O. In a lower frequency region, a portion of the 1115(+) cm^−1^ band could be downshifted to 1106 cm^−1^, and the 1086(−) and 1061(−) cm^−1^ bands were weakened in intensity. The frequencies of the bands at 1242(−)/1211(+) cm^−1^ in H_2_O were unclear; these bands might be also related to the vibrational modes of Thr sidechain (COH bending)^31^.

In contrast to the Na^+^-induced spectrum, fewer IR features were observed in the difference spectra of “20 mM -*minus*- 0 mM” LiCl, KCl, RbCl, or CsCl (traces-c. to -f., Fig. 5A). Overall, monovalent alkali metal cations other than Na^+^ that were tested shared similar IR features, as observed in the amide I (1665−1620 cm^−1^), amide II/carboxylate ν_as_ (1560−1550 cm^−1^) and carboxylate ν_s_ (1430–1390 cm^−1^) regions. Below 1300 cm^−1^, almost no signals were observed, except for the slight hint of a positive band at 1110 cm^−1^ in the lithium ion (Li^+^)-induced spectrum. The results suggested that the stator complex of PomAB possesses Na^+^-selective and non-selective cation binding sites. To extract Na^+^-selective IR features, the double difference of “Na^+^-K^+^” was calculated (upper trace, Fig. 5B). The carboxylate ν_s_ modes appeared at 1425(+), 1417(−), and 1406(+) cm^−1^, accompanied with the ν_as_ modes at 1554(−), 1545(+), 1537(−), and 1525(+) cm^−1^.

To examine the association of the Na^+^-selective IR bands with cell motility and/or ion transport, Na^+^- and K^+^-induced ATR-FTIR spectra were also measured in the motility impaired PomA-T158A, PomA-T186A, and PomB-D24N mutants. The double differences of “Na^+^−K^+^” were also calculated. As shown in Fig. 5B (middle traces), all the mutants showed basically featureless flat lines in the double difference. This was because in the three mutants the Na^+^- and K^+^-induced spectra exhibited IR profiles characteristic of cation binding to the ‘non-selective’ site (see Fig. S2). The results confirmed that the Na^+^-selective IR bands in WT PomAB could be associated with motility activation as well as Na^+^-selective cation transport. Since there are no carboxylic residues other than PomB-D24 in the TM region, the IR bands due to carboxylate in the double difference could be confidently assigned to the PomB-D24 sidechain. The H/D-sensitive positive band at 1115 cm^−1^ could be tentatively assigned to the C-O stretch mode^31^ of PomA-T158 and/or PomA-T186 sidechain, which could possibly coordinate to the Na^+^ ion.

Next, the effects of a motility mutation located in the periplasmic loop between TM-1 and TM-2 were examined. *V. alginolyticus* that expressed PomA-D31C displayed a slow motility phenotype with a threshold NaCl concentration of 38 mM^32^. Similar IR measurements revealed that PomA-D31C exhibited an Na^+^-induced spectrum with IR features similar to those in the WT, in addition to a minor positive at 1674 cm^−1^, even at a NaCl concentration below the motility threshold (see the lower panel in Fig. S2). In contrast, the K^+^-induced spectrum lost the w-shaped changes at 1650–1620 cm^−1^, which are characteristic of cation binding to the ‘non-selective’ site. As a result, the double difference of “Na^+^−K^+^” reproduced most of the Na^+^-selective features (magenta, lower trace in Fig. 5B), suggesting that the PomA-D31C mutation did not affect the Na^+^ binding to the Na^+^-selective site. The result supports that at least one of the “non-selective” sites could likely exist in the peripheral loop region.

Finally, the binding affinity of Na^+^ to WT PomAB was estimated using ATR-FTIR measurements. The IR intensities of peak/troughs, which are relevant to the Na^+^-selective features, at 1662(+)/1648(−) cm^−1^ and 1115(+)/1061(−) cm^−1^, were plotted against NaCl concentration. The data were fit by the Hill equation with parameters of dissociation constant K_d_ and the Hill coefficient values; 3.5 mM and 0.9 for the 1662(+)/1648(−) cm^−1^ and 6.5 mM and 0.7 for the 1115(+)/1061(−) cm^−1^, respectively (Fig. S3). The obtained binding affinity in the range of several mM was slightly smaller than the estimation obtained using the motility assay (half maximal effective concentration at 16 mM, calculated from reported data^32^).

### MD simulations

**Figure 6:**
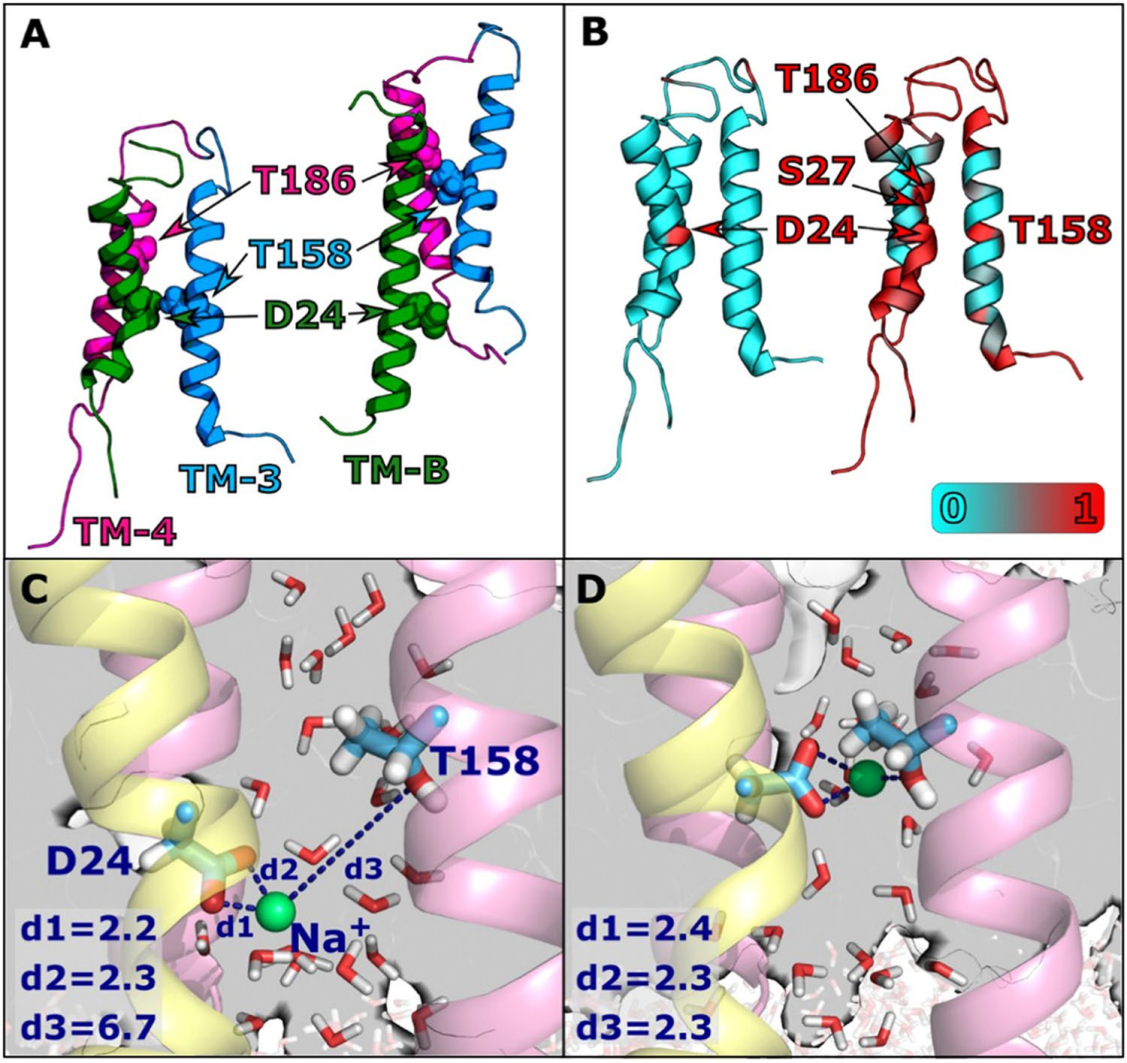
MD simulation of the Na^+^ binding residues of the stator complex. (**A**) Channels 1 (left) and 2 (right) after 400 ns of the WT MD simulation. The three chains (TM-3, TM-4, and TM-B) are shown in blue, pink, and green, respectively. T158, T186, and D24 are represented as spheres. (**B**) Contact map for Na^+^ (left) and water (right). Channel residues (Cα) are colored according to the fraction of time during the simulation in which they form an interaction with a Na^+^ ion or a water oxygen atom. An interaction between a protein residue and an atom is defined as any (non-hydrogen) atom of the residue residing within 3.5 Å of the Na^+^ ion or water oxygen. Results were calculated using simulation frames saved every 1 ns from the last 375 ns of the 400 ns WT MD simulation. (**C**) and (**D**) Distances (d1, d2, d3, in Å) of Oδ1 -D24, Oδ2 -D24 (TM-B, rendered as sticks on yellow ribbon), and Oγ1 -T158 (rendered as sticks on pink ribbon) to the nearest Na^+^ ion (green sphere), after 105 ns (**C**), and after 106 ns (**D**) of the WT MD simulation. Water molecules in the vicinity of the above-mentioned residues are displayed as sticks.

The stator complex of PomAB contains two channels that are topologically equivalent by C2 symmetry (hereafter designated as channel 1 and channel 2)^33^. Each is created by a PomA-PomB pair. The two remaining PomA units do not form channels. We observed the behavior of Thr158, Thr186 (PomA), and Asp24 (PomB) during a 400 ns MD simulation. During the simulation, a structural change occurred in channel 1, during which TM-3 and TM-4 slid toward the cytoplasmic side. This sliding motion located D24 and T158 at a distance of ~8 Å in channel 1, compared to ~19 Å in channel 2 (Figs 6A and S4A,B). In channel 2, sliding occurred to a lesser extent at the very beginning of the simulation, increasing the distance between D24 and T158, after which the distance remained constant.

During the MD simulation, a Na^+^ ion (inserted in the simulation box to maintain ionic strength and total charge) moved toward D24 in channel 1 and remained in the vicinity throughout the entire simulation. Among the channel residues, D24 had a unique affinity to Na^+^, implicating it as a pivotal ion binding site (Fig. 6B, left). Such an interaction did not occur in channel 2 (Table S1), further highlighting the asymmetry between the two channels. The Na^+^ ion entered from the cytoplasmic side and was located between D24 and T158, which came closer due to the sliding of PomA (Fig. 6C). Most of the time, Na^+^ was located at a distance of ~7 Å from T158 (Oγ1), but occasionally the distance shortened to <3 Å allowing the direct ligation of Na^+^ by both D24 and T158 (Fig. 6D). Na^+^ is additionally coordinated by water molecules (three in Fig. 6C and two in Fig. 6D), maintaining a constant coordination shell of five ligands. Analysis of the water affinity for the channel residues (Fig. 6B, right) clearly revealed that D24, T158, and T186, as well as S27 of PomB in channel 1 had a high affinity for water (1.00, 1.00, 0.98, and 0.95, respectively) compared to other residues in the channel.

Simulations for the mutants T158A and T186A were performed from two initial structures taken from frames of the WT MD simulation. For each mutant, one simulation commenced from the WT structure simulated for 2 ns (T158A-2, T186A-2) and the other for 50 ns (T158A-50, T186A-50). In the latter case, the structure change in the WT had already begun. The mutant simulations were performed for another 200 ns. In the T158A-2 and T186A-2 simulations, chain sliding did not occur in channel 1 and the D24-T158 distance remained similar to the initial distance of 14 Å (Fig. S4A, red and green lines). For T158A-50 and T186A-50, chain sliding did occur and the D24-T/A158 distance was shortened, as in the WT (Fig. S4A, purple and light green lines). However, for T158A-50, once the D24-A158 distance started to shrink, the Na^+^ ion near D24 totally escaped from the vicinity of the protein and did not rebind to D24 for a long period of time (it returned only at the end of the 200 ns simulation, when the D24-A158 distance started to slightly grow; see Fig. S4C,D). This behavior suggested that the configuration in which D24 and residue 158 are in close proximity might be unstable for Na^+^ binding when residue 158 is mutated to Ala. No compensating chain sliding in channel 2 was observed for the mutant in the simulations (Fig. S4B).

Regarding the water affinity in the mutants, D24 remained a major site for water docking. For channel 1, residue 158 displayed lower affinity only when it was mutated to Ala. Residue 186 showed reduced affinity only when a structural change occurred (T158A-2 and T186A-2), regardless of whether the change was to Ala or Thr. The complete water affinity data are presented in Table S1.

**Figure 7:**
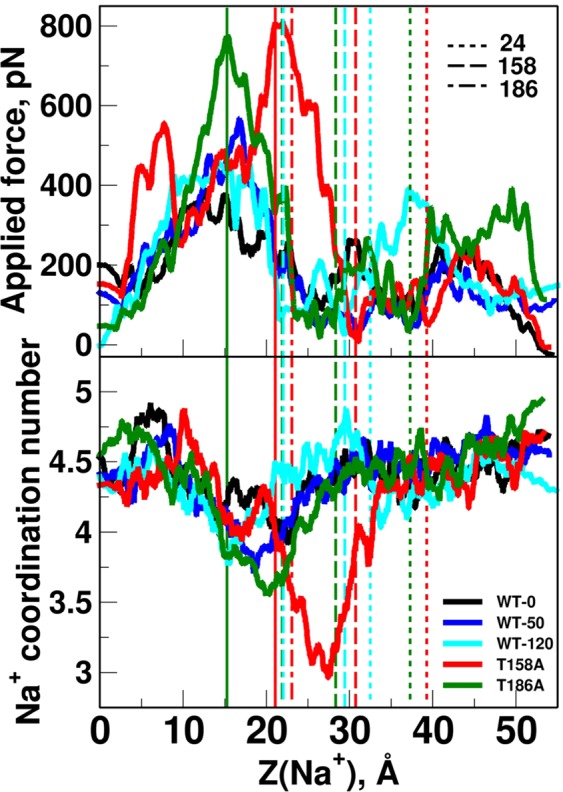
SMD simulation of Na^+^ ion moving in the ion channel. Top: applied force during the SMD simulation vs. z coordinate of moving Na^+^ ion. Bottom: Coordination number of Na^+^ vs. its z coordinate. Coordination number is considered as the number of polar atoms (O, N, S of protein and water) within 2.5 Å of Na^+^. For both panels: Direction of movement is from left to right. Each line consists of data assembled from all 10 simulations of each set. Individual SMD profiles are presented in Fig. S5A. For clarity of vision, each data point is calculated as an average over its 500 (top) and 100 (bottom) surrounding data points. Data before averaging is presented in Fig. S5B. Data averaged with increasing number of SMD trials (1, 2, 3, 5, 8, and 10) is presented in Fig. S6. Average z coordinates of T/A158, T/A186, and D24 (Oγ1 for Thr, Cβ for Ala, and Oδ1 for Asp) during the simulations are marked as dashed vertical lines (for SMD-WT-120 and the mutants’ simulations). Numbers next to the dashed lines in the legends mark the residue numbers D24, T/A158, and T/A186. Lines for average z coordinate for T/A186 in WT-120 and T186A runs (3^rd^ and 4^th^ lines from the left) slightly overlap. Locations of the maximal force peak for T158A and T186A simulations are marked as solid vertical lines.

The channel transport of Na^+^ was investigated using a series of steered MD (SMD) simulations. Three sets of simulations were conducted for the WT (SMD-WT-0, SMD-WT-50, and SMD-WT-120), and one series for each mutant (SMD-T158A and SMD-T186A), as detailed in the Methods section. Figure 7 (top) shows the applied instantaneous force versus the location of Na^+^, united for all runs in a set and smoothed. The former is the force required to pull the ion in the set velocity. Compared to the WT simulation, the maximal force applied in the mutant simulations was considerably larger than that for the WT (1.42 and 1.36 times higher than the highest peak for the WT for T158A and T186A, respectively). Figure S5A, and S5B present raw (non-averaged) data for each of the individual SMD trials. There, it is apparent that the mutant curves show larger peaks than the WT curves. Moreover, Fig. S6 shows convergence of the SMD plots as more trials (1, 2, 3, 5, 8, and 10) are added to the average. The current simulations were performed in a high velocity of the ion which does not allow accurate calculation of free energy along the path. However, the obtained results can be used for comparing the relative profiles of the WT and the mutants. We thus predicted that in the case of the mutants, the increased force peaks would translate to a large energy barrier along the channel, which would serve as a bottleneck for Na^+^ transport.

Slightly after the increase in the force peaks in the mutant SMD simulations, a large decrease in Na^+^ coordination number was observed for the mutants, especially T158A (Fig. 7, bottom). This drop also occurred in the WT, albeit to a smaller degree, and was possibly related to the close proximity of the narrowest part of the channel. In the case of the mutant, however, Thr was replaced by Ala, which should widen the path and presumably make more room for solvation shell atoms. Yet, the drop in the coordination number for the mutants was larger than that for the WT. Figure S5C shows coordination numbers separately for protein atoms (i) and water (ii). It is observed that all runs except T158A gain a water coordination upon passing near T158 (replacing a prior protein coordinating atom). In T158A a water is lost (in addition to the common loss of protein atom), causing an overall loss of two coordinating atoms. In the T186 site, only one water molecule is lost, resulting in a smaller negative peak for T186A.

## Discussion

We investigated the function of the Thr residues for ion translocation in the flagellar stator complex. The Thr residues are well preserved in the TM regions of the stator complex in the Na^+^ driven motor. Thr residues of PomA-T158, PomA-T185, and PomA-T186 were essential for the motor function and ion translocation. Among them, the PomA-T185A mutation affected the formation of the complex between PomA and PomB. ATR-FTIR analysis revealed that the PomA-T158A and PomA-T186A mutants lost the binding signal by Na^+^, similar to the PomB-D24N mutant. These residues may form a Na^+^ binding site in the stator complex. A previous ATR-FTIR study of PomA/PomB, in which spectra were measured at pH 5.5, revealed that Na^+^-binding is accompanied by deprotonation of carboxylic acids, including PomB-D24^30^. One problem in the previous study was that we observed cation binding FTIR signals not only for Na^+^, but also for all monovalent cations, which is contradictory to the cation selectivity observed in the motility experiments. In contrast, we clearly observed Na^+^-specific binding signals in this study, which reflect strictly the motor performance.

It has been suggested that *V. cholerae* PomB-S26 (S27 in *V. alginolyticus*) is the second most important residue besides *V. cholerae* D23 (D24 of *V. alginolyticus*) in the PomB channel^20^. S26 may work together with D23 to perturb the hydration shell of Na^+^ for its translocation through the stator channel. In the MD simulations it showed high water affinity supporting the above claim. However, it had been unclear whether a single mutation, except for the Asp residue of PomB, in the TM region of the stator complex can produce the non-motile phenotype. The residue is highly conserved as Ser or Thr in either the proton- or the sodium-driven type (Fig. 1). Thus, the Ser residue may not specific for the sodium ion.

The proton-specific Bs-MotAB and the sodium-specific Bs-MotPS were changed into the sodium-specific and the proton-specific type, respectively, by the triple mutation around the periplasmic boundary of the TM-B region^8^. Based on the alignment, two mutants were constructed. The sequences of VLYASSS from Bs-MotB and LLFSMSQ from Bs-MotS were changed to LLYSSSQ and VLFAMSS, respectively. The Val, Ala, and Ser (VAS) residues were exchanged with Leu, Ser, and Gln (LSQ) in subunit B. This region seems to correspond to the entrance of the ion in the stator channel. The LLLSFSE sequence from *V. alginolyticus* corresponds to those from *B. subtilis*, and the side chain of the amino acids (LSE) displays greater similarity to the sodium type of Bs-MotS. The Ser (S38) of LSE in *V. alginolyticus* PomB, which is located two residues away from the conserved Ser (S40 of PomB), is close to the D170 of TM-A3 derived from PomA^13^. This region may face the TM-A4 region positioned around T185 or T186 of PomA (Fig. 1).

The WT complex may possess multiple cation binding sites, which are Na^+^-selective and non-selective. The Na^+^ ions are supposed to bind at the both sites, therefore the double difference spectrum of “Na^+^− K^+^” should represent the structural changes in the former binding site. Since PomA-T158A, PomA-T186A, and PomB-D24N lost the Na^+^-selective IR features in the double difference, the three residues likely cooperatively coordinate to the Na^+^ ion and/or its solvation water molecule in the Na^+^-conducting pathway. On the other hand, these mutations did not affect the cation binding to the non-specific site. Thus, the latter site can be located in the periplasmic and/or cytoplasmic domains. Since the PomA-D31C mutation produced the opposite effects, cation binding to the two sites appears to be relatively independent, with each site having different physiological roles.

MD simulations of the stator complex model were conducted to investigate the role of the T158 and T186 residues from PomA and the D24 residue from PomB. During the simulation, a Na^+^ ion originating from the solution was permanently docked near D24, further supporting the hypothesis that the residue serves as an ion binding site. Due to the strong binding between Na^+^ and D24, it is unlikely that the ion would further enter the channel during an unbiased MD simulation, thus the lack of binding affinity in T186, located further above, does not rule it out as a Na^+^ binding site. In the same channel where Na^+^ docking occurs (channel 1), the distance between the allegedly crucial residues shortened due to the sliding of PomA toward the cytoplasmic side. Structure change in channel 1 occurred after Na^+^ binding, suggesting that TM sliding was enhanced by the ion binding. MD simulations for the mutants T158A and T186A did not show a spontaneous vertical sliding of PomA (T158A-2 and T186A-2). However, after some structural changes (T158A-50 and T186A-50), further chain sliding was inevitable. However, even when the structural change occurred, binding of Na^+^ did not occur if T158 was mutated to Ala. These results suggest that the three-way interaction (D24-Na^+^ -T158) achieved by the chain sliding is unique to the WT motion.

Chain sliding only occurred in one of the two channels. Subunit asymmetry was reported to be essential for function in other molecular machines such as the Vacuolar-type ATPase^34^ as well as the ExbBD complex in the gram-negative bacteria Ton system^35,36^. The latter system, for which crystal and Cryo-EM structures were recently solved, exhibits sequence homology with MotAB/PomAB. Although the overall symmetry of the ExbBD complex differs from that of PomAB (ExbB and ExbD ratio of 6:3), the channel architecture is common for both systems, consisting of two TM helices of ExbB/PomA and the single helix of ExbD/PomB. Interestingly, in ExbBD a similar trio of two threonines (T148 and T181 of ExbB) and an Asp residue (D25 of ExbD) are located in strategic points and speculated to play a role in proton conductance. Fig. S7A (left) presents the aligned structures of a single channel of ExbBD (between chains A and I) and PomAB, showing that the relative locations of the Thr pairs are similar. Alignment of the channel regions of ExbB and PomA yields an identity of 27% and similarity of 50% (Fig. S7B), moreover the locations of both Thr pairs are conserved. For ExbD and PomB no significant sequence identity was found, however, the corresponding Asp residues are located in strikingly similar positions. ExbBD also exhibits asymmetry where each subunit is slightly shifted with respect to the adjacent subunit. Similar to the conformations observed during MD simulations of WT PomAB, the threonines and the Asp in the ExbBD structure are located at slightly different relative distances at each of the three channels (Fig. S7C). This property was speculated to be important for the proper utilization of the proton motive force in ExbBD^36^. Figure S7A (right) presents an alignment of a different ExbBD channel (chains D and G) against channel 1 of PomAB after 105 ns of simulation, in which chain sliding has occurred. It is apparent that the structure of the DG channel is more similar to the shifted PomAB channel 1 than to the initial PomAB structure (Fig. S7A, left).

The T158, T186, and D24 residues featured a notably high water affinity that was reduced in the mutants. The ability of those residues to form hydrogen bonds with water molecules, combined with their favorable positions along the channel, make them good candidates as water docking sites. Water molecules along the channel serve as a solvation shell for the transported ion, and the existence and location of such sites might be crucial for the channel function. In the SMD simulations, a significantly larger force was required to transfer the ion through the channel for the mutants. The peak in the applied force might correspond to a free energy barrier, which might be large enough to completely block further ion transport down the channel. This is consistent with the complete loss of ATR-FTIR signal for Na^+^ near D24 in the mutants. As stated earlier, since the pulling speed used in the current procedure was high, we have to require attention not to overinterpret the obtained results. The absolute energy values cannot be calculated from the current SMD calculations. However, based on the results we can predict which barrier is high for transporting Na^+^ ion through the channel between WT and the mutants. In the present study, the pulling simulations were conducted with similar conditions for all systems and we observed a notable difference between the WT and the mutant simulations. Furthermore, the simulations in the current work were performed based on a modeled structure. We have to recognize that a modeled structure is not always as reliable as an experimentally solved structure. We therefore provided an extended quality assessment of our model. The main observations from the MD calculations is that T158 and T186 are crucial for Na^+^ transport. These were concluded by observing the mutant simulations while using the WT simulation as a reference, and avoiding the calculation of direct measurable quantities. Finally, with the current simulation setup (system size and complexity and simulation time) it is not feasible to directly simulate the dynamics of the Na^+^ ion in real-time. The results from the MD simulations show that from a structural point of view, Asp24 is the (only) favourable location for ion binding.

In Na^+^-translocating V-ATPase from *Enterococcus hirae*, the X-ray crystal structure of the Na^+^-bound form has been resolved^24^. The Na^+^-induced ATR-FTIR spectra were interpreted as indicating that the protonated carboxylic acid, E139, in the free form was deprotonated to ligate the cation^37^. Based on the frequency separation of ν_as_−ν_s_ at 139 cm^−1^ in the Na^+^-bound form, the authors discussed that the Na^+^ ion was ligated by E139 with a pseudobridged mode, in accordance with the crystal structure. In PomAB, the corresponding ν_as_−ν_s_ values were obtained as 100–139 cm^−1^ (depending on the combination of 1545/1525 cm^−1^ as ν_as_, 1425/1406 cm^−1^ as ν_s_) in the Na^+^-selective site, and 159–164 cm^−1^ (depending on the metal species) in the non-selective sites. This indicated that the Na^+^ ion is ligated by PomB-D24 in the Na^+^-selective site with a bidentate or pseudobridged mode, while at the non-selective site the cation is ligated by a carboxylate with less bidentate character, based on published criteria^38,39^. The MD simulation resulted in a model structure of Na^+^ coordination with a bidentate mode in D24 carboxylate in the Na^+^-conducting pathway, which agrees with the IR observation.

Many reports have established that the hydroxyl group of Thr contributes to a Na^+^ binding site in the transmembrane regions. In F-type ATP synthase of *Ilyobacter tartaricus*, the Na^**+**^-binding site is provided by Q32, V63, E65, and S66. In addition, Thr (T67) coordinates the key water ligand (but not Na^+^) for Na^+^-binding site^23^. In the ATPase of *Acetobacterium woodii*, the Na^+^ binding site is formed by E62 (TM2 helix), Q29 (TM1), V60 (TM2’), and T63 (TM2’)^25^. In the V-ATPase from *Enterococcus hirae*, the Na^+^-binding site consists of the side chains of T64 (H2 helix), Q65 (H2), Q110(H3), and E139 (H4), as well as the main-chain carbonyl of L61(H2)^24^. In PutP, Na^+^/proline transporter, T341 is crucial for Na^+^ binding^40^. In LeuT, a bacterial homologue of Na^+^/Cl^−^ dependent transporters, the first Na^+^ binding site is provided by its substrate, leucine, and its amino-acid residues, A22 (TM1), T254 (TM6), N27 (TM1), N286 (TM7), and T254 (TM6)^22^.

**Figure 8:**
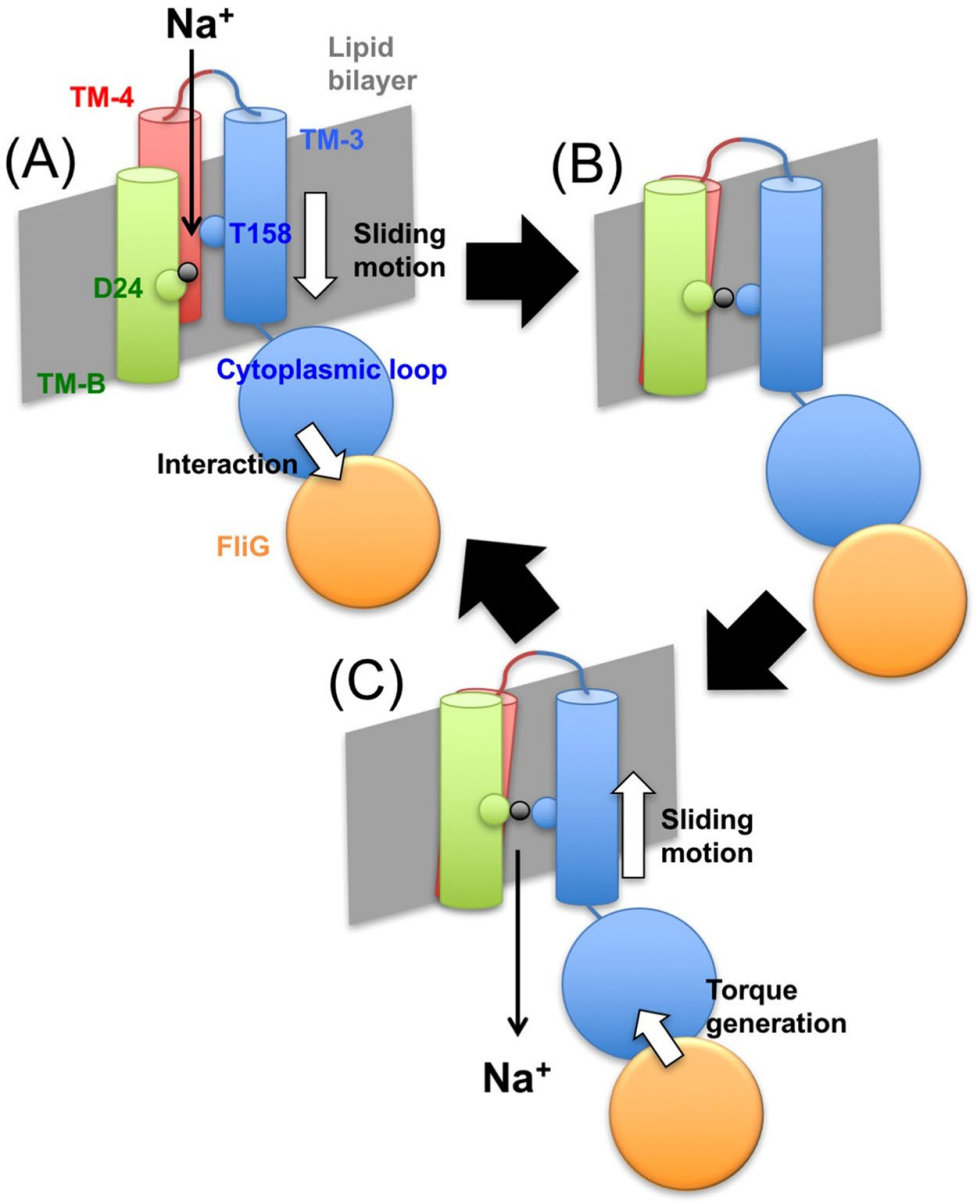
A mechanochemical model for the flagellar motor. Interaction of the cytoplasmic loop in PomA with FliG induces sliding motions of TM-3 and TM-4 toward cytoplasmic side (**A**). This rearrangement approaches T158 in TM-3 toward D24 in TM-B and forms Na^+^ binding site by three modes of interaction (**B**). After release of the Na^+^ into the cytoplasmic side, TM-3 and TM-4 return to the original position. This re-sliding motion induces a conformational change in the cytoplasmic loop to produce torque at the interface with FliG (**C**).

In the MD simulation, we observed transient interaction of side chain of Thr158 with Na^+^ by three-way interaction (D24-Na^+^-T158). We have previously proposed that interaction between PomA and FliG is a cue to transport Na^+^ through the stator complex^41,42^. According this hypothesis, we propose transient three-way interactions are stabilized by the stator-rotor interaction. The rotor, FliG interacts with the cytoplasmic loop of PomA located between TM2 and TM3. This interaction could induce sliding motion of TM3 as observed in MD simulations (left figure in Fig. 6A) in one of the channels. In this model, the structure facilitates sodium ion-binding in D24-PomB and T158-PomA by the three-way interactions (Fig. 8). This three-way interactions might be a gate of sodium ion-translocation that lowers the energy barrier as shown by SMD simulation (Fig. 7). After translocation of sodium ion, slided TM3 returns to the original position. This sliding motion to the original position may generate torque between the stator and the rotor. This is molecular mechanism of the mechanochemical cycle of the bacterial flagellar motor. We calculated the coordination number of Na^+^ along its path in the channel. In the mutants, a large decrease in Na^+^ coordination number that occurred slightly after the increased force peaks was evident. This observation may hint at the molecular mechanism of the T158 and T186 function. We suggest that the polar groups of the Thr residues interact with Na^+^, either directly or via its solvation shell. In such case, the lack of polar groups for the mutants will hinder this favorable interaction and consequently the smooth transport of the ion. Na^+^ coordination profiles (Fig. S5C) suggest that while T186 interacts directly with Na^+^, T158 interacts exclusively via bridging water molecules.

## Experimental Procedures

### Bacterial strains, plasmids, and culture medium

Bacterial strains and plasmids used in this study are listed in Table S2 ^43–46^. The plasmid for expression of PomA and PomB in *E. coli* was constructed as follows. The plasmid, pET-22b(+)-pomAB-his, which was designated pTSK6, was digested by the restriction enzymes NdeI and BamHI, and ligated into the pCold4 vector at the same sites. This plasmid can express PomA and PomB with the His6-tag at the C-terminal PomB under cold shock. Single point mutations in *pomA* and *pomB* were generated by QuikChange site-directed mutagenesis (Agilent) according to the manufacturer’s protocol.

*E. coli* cells were cultured in LB [1% (w/v) Bacto-tryptone, 0.5% (w/v) yeast extract and 0.5% (w/v) NaCl]. *V. alginolyticus* cells were cultured in VC medium [0.5% (w/v) polypeptone, 0.5% (w/v) yeast extract, 0.4% (w/v) K_2_HPO_4_, 3% (w/v) NaCl, and 0.2% (w/v) glucose] or VPG medium [1% (w/v) polypeptone, 0.4% (w/v) K_2_HPO_4_, 3% (w/v) NaCl, and 0.5% (w/v) glycerol]. Ampicillin was added to a final concentration of 50 μg/ml for *E. coli*. Chloramphenicol was added to final concentrations of 2.5 and 25 μg/ml for *V. alginolyticus* and *E. coli*, respectively.

### Amino acid sequence analysis

Multiple sequence alignments of amino acid sequence of PomA or PomB from Na^+^-driven motor were performed using CLUSTAL Omega^47^. Predictions of transmembrane regions in PomA and PomB from *V. alginolyticus* were performed using TOPCONS^48^.

### Motility assay

One microlitre of overnight cultures of *V. alginolyticus* NMB191, carrying the plasmid pHFAB or the vector pBAD33 at 30 °C in VC medium were spotted onto VPG soft-agar plates [VPG medium and 0.25% (w/v) Bacto agar] containing 0.02% (w/v) arabinose. The plates were incubated at 30 °C for 5 h.

For the analysis of bacterial swimming, these overnight cultures were diluted 100-fold into fresh VPG medium with 0.02% (w/v) arabinose. After incubation of these cultures at 30 °C for 4 h, cells were diluted 100-fold in VPG medium and were observed using dark-field microscopy. Movies of the cell motility were recorded on PC (Power director, CyberLink) and the swimming fractions and speeds of the cells were measured using software for motion analysis (Move-tr/2D, Library Co.).

### Growth curves

Growth curves to investigate Na^+^-transport activity were conducted by a slight modification of the procedure published previously^27^. Briefly, overnight cultures of *E. coli* DH5α, carrying the plasmid pTSK37 or the vector pBAD33 at 37 °C in LB were diluted into fresh LB with 0.2% (w/v) arabinose at an optical density at 660 nm (OD_660_) of 0.02. Cells were grown at 37 °C for 8 h and the OD_660_ was recorded every hour.

### Detection of proteins by Western-blotting

Western-blotting was performed as previously described^49^, except that anti-PomA antibody (PomA91)^43^ or anti-PomB_C_^50^ was used as the primary antibody.

### Expression of PomAB and preparation of membrane fraction

*E. coli* harboring the PomAB expression vector (pCold4-pomApomB-His6) were cultured overnight in LB at 37 °C. After 100-fold dilution into fresh LB, the fresh culture was incubated at 37 °C for 2.5–3.0 h until the OD_660_ reached to 0.4–0.6. The culture was transferred to ice-water for 30 m and isopropyl β-D-1-thiogalactopyranoside (IPTG) was added at a final concentration of 0.5 mM. The culture was incubated at 16 °C for longer than approximately 20 h.

Cells were collected by centrifugation and suspended in 50 mM sodium phosphate buffer (pH 8.0) and 200 mM NaCl. These samples were passed through a model 5501-M French Press (Ohtake Works) twice for large scale culture or sonicated (Sonifier 250; Branson) three times for 30 sec each time for small-scale culture. Disrupted cells were centrifuged (17,000 × *g* for 10 min at 4 °C) to remove unbroken cells and the supernatants were ultra-centrifuged (150,000 × *g* for 30 min at 4 °C). The pelleted samples were homogenized in the same buffer and stored at −80 °C until use.

### Pull-down experiment

The experiment was performed at room temperature except centrifugation steps. DMNG was added to 500 μl of the membrane fractions at final concentration of 0.5% (w/v) and the mixtures were incubated for 30 min. These samples were centrifuged (150,000 × *g* for 30 min at 4 °C) and their supernatants were mixed with 50 μl of Ni-NTA agarose (QIAGEN) pre-equilibrated in Wash buffer [50 mM sodium phosphate buffer (pH 8.0), 200 mM NaCl, 10 mM imidazole, and 0.05% (w/v) DMNG] and incubated for 1 h. After centrifugation (2,000 × *g* for 30 sec at 4 °C), the supernatant was removed and 500 μl of Wash buffer was added to the pelleted agarose and incubated for 10 min. This step was repeated one more time. After centrifugation (2,000 × *g* for 30 sec at 4 °C), the supernatant was removed and 500 μl of Elution400 buffer [50 mM sodium phosphate buffer (pH 8.0), 200 mM NaCl, 400 mM imidazole, and 0.05% (w/v) DMNG] was added and incubated for 30 min. After centrifugation (2,000 × g for 30 sec at 4 °C), the supernatant was collected. Membrane fractions and final eluted proteins were analyzed by Western-blotting.

### Purification of the complex of PomAB

Most of procedures were performed at room temperature unless indicated. DMNG was added to a final concentration of 0.5% (w/v) to the membrane fraction in which PomA and PomB were overexpressed, and the mixture was stirred at 30 °C for 30 min. After centrifugation of the sample (150,000 × g for 30 min at 4 °C), the supernatant was mixed with TALON metal affinity resin (Clontech) pre-equilibrated with Wash buffer and stirred gently in a cold room (4 °C) for 1 h. The mixture was loaded to an open column and washed with three column volumes of Wash buffer. Then, two column volumes of Elution buffer [50 mM sodium phosphate buffer (pH 8.0), 200 mM NaCl, 200 mM imidazole, and 0.05% (w/v) DMNG] was applied and eluted samples were collected. Peak fractions confirmed by SDS-PAGE were collected, mixed with 2-mercaptoethanol at a final concentration of 0.1% (v/v), and concentrated to approximately 500 μl using an Amicon Ultra-4 MWCO 100,000 filter (Millipore). The sample was centrifuged (150,000 × g for 10 min at 4 °C) and applied to an Enrich SEC. 650 10 × 300 column (Bio-Rad) using the AKTA explore system (GE Healthcare) at a flow rate of 0.75 ml/min. Eluted samples were confirmed by SDS-PAGE and peak fractions were concentrated as before without addition of 2-mercaptoethanol. The protein concentration was determined by the Pierce BCA method (Thermo Scientific) using bovine serum albumin as the standard.

### ATR-FTIR spectroscopy

For ATR-FTIR measurements, purified and detergent-solubilized PomAB proteins of the WT and mutants (PomA-T158A, PomA-T186A, PomA-D31C, and PomB-D24N) were reconstituted into a lipid mixture of 1-palmitoyl-2-oleoyl-sn-phosphatidylethanolamine (POPE) and 1-palmitoyl-2-oleoyl-sn-phosphatidylglycerol (POPG) at a molar ratio of protein complex (PomA)_4_(PomB)_2_: lipid = 1:40. The detergent molecules were removed by incubation with Bio-beads SM-2 (Bio-Rad), and the lipid-reconstituted proteins were collected by centrifugation. After several wash/spin cycles using a buffer (10 mM MOPS-Tris, pH 7.0, 5 mM MgCl_2_), an aliquot of sample suspension was placed on the surface of a three-reflectance Si ATR crystal (DuraSampleIR; Smith Detection). After it was dried with a gentle stream of N_2_ gas, the dry layer was rehydrated by the flow of perfusion buffer (10 mM MOPS-Tris, pH 7.0, 5 mM MgCl_2_) at a flow rate of 0.4 ml/min. The temperature of flow cell was maintained at 20 °C. The ATR-FTIR spectra were recorded at 2 cm^−1^ resolution using an FTIR spectrophotometer equipped with a liquid nitrogen-cooled MCT detector (Agilent). For the measurement of Na^+^-induced difference spectra, typically a background spectrum (260 interferograms co-added for 5 min) was measured with the sample under a flow of NaCl-free buffer, and then the buffer was switched to one containing 20 mM NaCl. When equilibrated, the sample spectrum was recorded. The forward/backward (Na^+^-binding/unbinding) measurements were repeated, and the spectra were averaged for 6–30 cycles to produce a raw spectrum as “20 mM -*minus*- 0 mM” NaCl. The final spectra were calculated by subtraction from the raw spectra by baseline drifts due to protein swelling/shrinkage and free salt/buffer ions in the external buffer, when necessary. In the D_2_O medium, the buffers were prepared at pD 7.0, applying an empirical conversion formula (pD = pH_meter reading_ + 0.4)^51^. To examine the metal selectivity, the buffer containing 20 mM LiCl, KCl, RbCl, and CsCl were used instead of NaCl. For the binding assay, the concentrations of NaCl were 0.5, 2, 10, 20, 50, and 100 mM.

### MD simulations

The TM regions of the PomA/PomB complex were modeled based on the MotA/MotB 3D structure^19^ using the Modeller 9.11 program^52^, by mapping the PomA/PomB sequence^53^ onto the MotA/MotB structure. The residues included in the current model are 1–59 (TM-1 and TM-2 of PomA and part of the loop connecting them) and 144–205 (TM-3 and TM-4 of PomA and part of the loop connecting then), and 12–42 for PomB. The sequence of PomA/PomB was mapped onto the MotA/MotB structure according to the alignment in a previous report^53^. The sequence identity between PomA/PomB and MotA/MotB was 24% for the modelled regions. 10 models were generated and the best model according to the DOPE and GA341 scores was chosen. The structure of MotA/B used for modeling of PomA/B was the best structure (the one with the smallest violations of the S-S restraints) achieved after “Evaluation & selection” stage in Fig. S1 of the paper^19^, which was conducted for 100-ns after releasing initial restraints.

The structural variability of the initially generated 10 models was assessed. The 10 created models were sufficiently similar to each other, the average RMSD value between all pairs was 1.93 Å, the maximal RMSD is between models 3 and 6 with a value of 2.36 Å. Regarding the model which was eventually used for the simulations (model 1), its RMSD to the other models vary from 1.83 Å to 2.19 Å. Seeing that the structural variation between the generated models was small, we used the best scoring model of the initial 10-member group, without further model generation.

The quality of the selected model was assessed using a program of Procheck^54^. The Ramachandran plot in Fig. S8 shows that close to 90% of the residues are located in the most favoured regions. The residues in disallowed regions are all located at the periplasmic loops, except for PomA-K49 which located at the boundary of an allowed region. Violations the Ramachandran plot in such loop regions are ascribed to the fixed rigid nature of the created model and are released during the MD simulations, as occurred in the present case (data not shown). Other main-chain and side-chain parameters are all within the acceptable range, as shown in Fig. S9.

Helix propensities were calculated for channel 1 and 2 (Figs S10–S12). Data are shown for the initial (modeled) structures of the MD production runs for PomAB and MotAB, as well as during the simulation for PomAB. Complementing Figs S10–S13 of channel 1 shows the location of the three transmembrane helices with respect to the membrane for the initial (modeled) structure (top), and for snapshots after 105 ns and 305 ns of the MD simulation (middle and bottom, respectively). Helical regions of the protein are colored in purple.

For residues 17–20 of TM-B (according to PomB sequence numbering) the helical structure is partly disrupted for channel 1 during the simulation. However, for Asp24 (where ion binding occurs), the helical structure is maintained throughout the whole simulation and the region is firmly embedded inside the membrane. For residues 35–42, located at the other end the initial modeled structure shows an unwound structure. In channel 2, the helical structure is regained throughout the simulation for the residues 35–39, whereas in channel 1 the helical structure is not regained. In the latter, this region is no longer embedded in the membrane due to upward sliding of the TM (Fig. S13, middle and bottom).

For TM-3 and TM-4, where structure change occurs, channel 1 and 2 show similar behavior. In the initial (modeled) structure, non-helical regions are seen embedded in the membrane (Fig. S13 top, residues 166–167 in Fig. S11, and residues 178–181 in Fig. S12). However, during the simulation the membrane-embedded residues regain their helical form so that there are no membrane-embedded non-helical regions. The initial structure was subjected to 7 ns MD with the protein fixed and then restrained before undergoing the 400 ns sampling MD simulation.

The gradual fitting of the helices to the membrane boundaries occur both for channels 1 and 2 (for the latter structure change was not observed), suggesting that initial partial unwinding of the helices does not play a critical role in the functional structural change observed herein. Therefore, although we do not underestimate the importance of proper modeling, we believe that in the current case it had little effect on the specific outcome. As part of our continuing work on the current system as well as other related systems for which experimental structures are not yet available, we are working on refining our model prediction methods as our next project and will hopefully be able to produce a revised model, for which our current assumptions will be re-examined.

The final PomA/PomB complex structure was subsequently embedded into a lipid bilayer containing the 324 POPE molecules and 108 POPG molecules at a ratio of 3:1 using the program g_embed^55^. The z-axis was set to be perpendicular to the membrane surface. The system was solvated in a water box consisting of 31,586 TIP3P water molecules, and Cl^−^ and Na^+^ ions were added to a 0.15 M to neutralize electric charge. The simulations were conducted under the assumption of neutral pH, aspartic acid and glutamic acid residues were unprotonated. There were no histidines in the model.

The NAMD program^56^ and the CHARMM36^57^ force field were used for the MD simulations. NBFIX parameters for Na^+^ and carboxylates were taken from the paper^58^. An increased LJ diameter used here is expected to reduce the overbinding of the Na^+^ ion to the lipid headgroups. After energy minimization for 1000 steps, the system was simulated for 1 ns with all the protein atoms fixed. Then it was simulated for 6 ns under restraints, starting from a force constant of 10 kcal mol^−1^ Å, which were gradually reduced. Finally, after all restraints were removed, data were sampled for 400 ns at constant temperature and pressure. Temperature was kept at 310 K by the Langevin thermostat, and the pressure was maintained at 1 bar using the Langevin piston. Non-bonded interactions were calculated with a cutoff of 14 Å and were evaluated using the particle mesh Ewald method. A timestep of 2 fs was used for integrating the equations of motion, and all bonds involving hydrogen atoms were kept rigid using the SHAKE algorithm. The PDB-formatted coordinates of the initial model, after 105 and 305 ns MD are provided as Supplementary Data.

The hydrophobic thickness of the membrane was calculated as the difference between the average z coordinates of the phosphorus atoms of the top and bottom leaflets. The calculated values were 38.2 ± 0.3 and 37.3 ± 0.2 Å, for the initial model and after 105 ns of simulation, respectively, conforming with previously calculated values^59^ of 36.0 ± 0.1 Å (for both POPE and POPG membranes).

The hydrophobic thicknesses of the TM helices were calculated to examine the degree of matching with the membrane thickness. The results are shown in Table S3 for the initial model WT structure and after 105 ns of MD simulation. Value were calculated for all TM chains and for each of the four subunits of PomA and two subunits of PomB. As seen in the data in the table, all values are within the range of 28.8 ± 0.2 and 38.2 ± 0.3 Å for the initial structure (26.9 ± 0.2 and 37.3 ± 0.2 Å for the structure after 105 ns MD), which are the calculated hydrophobic thicknesses as measured by difference in average z coordinates of phosphorus atoms and by these of the ester carbons of the lipids. Thus, both for the initial model and for the structure after 105 ns of MD, the border between the water-exposed and the hydrophobic regions lie between the phosphorus atom and the ester carbon of the lipid headgroups, showing that the hydrophobic thickness of the protein roughly matches this of the membrane.

The PomA cytoplasmic domain between TM-2 and TM-3 were not included in the current model. We calculated the distance between the last residue of TM-2 (Q54) and first of TM-3 (V149), which were included in the current model. For the initial structure of the WT the distances were 16.29, 19.69, 15.47, and 20.30 Å, for subunits 1, 2, 3, and 4, respectively (the channels where structure change occurred forms between subunit 2 of PomA and subunit 1 of PomB). After 105 ns of MD simulation without restraints the distances were 16.45, 16.81, 18.42, and 16.43 Å for subunits 1, 2, 3, and 4. Overall the PomA subunits which form channels (2 and 4) shorten the distance between the TM helices whereas the remaining subunit slightly increase the distance. However, the change in distance between the initial and the simulated structures is smaller than the subunit variations in the initial structure. Moreover, since the missing cytoplasmic loop is 94-residue long, it is expected to be quite flexible so that its absence in the current model is not expected to greatly affect the stability of TM parts. Last, the channel forms between TM-3, TM-4, and TM-B, specifically, the Na^+^ binding site is located in the triangular region between those three helices thus the distance between TM-2 and TM-3 is not expected to directly influence Na^+^ binding.

To examine the role of Thr158 and Thr186 of PomA, we conducted MD simulations for two mutant structures, T158A and T186A. These mutations were created separately and on all four subunits of PomA. For each mutant, two simulations were initiated with initial structures taken from frames of the WT trajectory, one set at 2 ns (T158A-2, T186A-2) and another set at 50 ns (T158A-50, T186A-50). The duration for the mutant simulations was 200 ns. All other MD parameters were similar to those in the WT simulation.

A SMD simulation^60^ was performed, in which the Na^+^ ion was pulled along the channel in a constant velocity. A spring force constant of 6.16 kcal mol^−1^ Å^−2^ was applied along the channel axis (z-axis) to pull the Na^+^ at a velocity of 0.05 Å/ps. In addition, in order to prevent the ion from escaping the channel by sideways motion, the x and y coordinates of the Na^+^ were harmonically restrained around their initial positions with a force constant of 1 kcal mol^−1^ Å^−2^. The Na^+^ ion was initially placed above the channel entrance (between the C-terminus of TM-B and the loop connecting TM-3 and TM-4) and traveled a distance of 50 Å during 1 ns.

For each structure, ten individual SMD simulations were performed, starting from slightly different structures, and their average was determined. The initial structures for the individual SMD simulations were taken from frames of the MD simulations. For the WT, three sets of SMD simulations were performed, with the initial structures taken from the MD simulation after {0, 1, …, 9 ns}, {50, 51, …, 59 ns}, and {120, 121, …, 129 ns}. For the mutants, one set was performed for each starting from {60, 61, …, 69 ns} of the MD simulation (T158A-2 and T186A-2).

## Supplementary information

Supplementary Tables and Figures

Dataset1
