# Supplementary material for: Essential ion binding residues for Na+ flow in stator complex of the Vibrio flagellar motor
Source: Sci Rep. 2019 Aug 2;9:11216. doi: 10.1038/s41598-019-46038-6 (PMC6677748; doi:10.1038/s41598-019-46038-6)
Supplement: Supplementary file 1 — Supplementary Tables and Figures [file 41598_2019_46038_MOESM1_ESM.pdf]

Supplementary information

Essential ion binding residues for Na^+ flow in stator complex of the *Vibrio* flagellar motor

Yasuhiro Onoue[1*], Masayo Iwaki[2*], Ai Shinobu[4*], Yasutaka Nishihara[3], Hiroto Iwatsuki[1], Hiroyuki Terashima[1], Akio Kitao[4], Hideki Kandori[2] and Michio Homma[1]

[1]Division of Biological Science, Graduate School of Science, Nagoya University, Furo-cho, Chikusa-ku, Nagoya 464-8602, Japan
[2]Department of Life Science and Applied Chemistry, Nagoya Institute of Technology, Showa-ku, Nagoya 466-8555, Japan
[3]Institute of Molecular and Cellular Biosciences, The University of Tokyo, 1-1-1 Yayoi, Bunkyo, Tokyo 113-0032, Japan
[4]School of Life Science and Technology, Tokyo Institute of Technology, 2-12-1 Ookayama, Meguro, Tokyo 152-8550, Japan

*These authors contributed equally to this study.

Correspondence to Michio Homma and Hideki Kandori:
g44416a@cc.nagoya-u.ac.jp (MH) and kandori@nitech.ac.jp (HK)

Table S1: Fraction of time during the simulation in which there is an interaction[a] between residue X and a Na^+ ion, or a water oxygen[b]

MD run and channel	Interaction of Na+ with residue X				Interaction of water with residue X			
	D24	T/A158	T/A186	S27[c]	D24	T/A158	T/A186	S27[c]
WT-channel 1	0.90	0.01	0	0	1.00	1.00	0.98	0.95
WT-channel 2	0	0	0	0	1.00	0.98	0.59	1.00
T158A-2-channel 1	0.97	0	0	0	1.00	0.21	0.39	0.93
T158A-2-channel 2	0.65	0	0	0	1.00	0.49	0.99	0.99
T158A-50-channel 1	0.11	0	0	0	1.00	0.81	0.98	0.99
T158A-50-channel 2	0.02	0	0	0	1.00	0.28	0.01	0.99
T186A-2-channel 1	0.98	0	0	0	1.00	0.96	0.58	0.91
T186A-2-channel 2	0.20	0	0	0	1.00	0.90	0.03	0.99
T186A-50-channel 1	0.97	0	0	0	1.00	0.97	0.94	0.95
T186A-50-channel 2	0.11	0	0	0	1.00	0.99	0.09	0.99

[a]An interaction was counted whenever a Na^+ ion/water oxygen was within 2.5 Å of any (non-H) atom of the residue

[b]Data for the calculation were taken in increments of 1 ns during the trajectory. The first 25 ns of the simulations were not included in the calculation

[c] PomB-S27

Table S2. Strains and plasmids used in this study

Strain and plasmid	Description	Source or reference
Vibrio alginolyticus strain		
NMB191	Δ*pomAB* Rif^r, Pof^+, Laf^-	43
Escherichia coli strains		
DH5α	Recipient for cloning experiments	
BL21(DE3)	Host for protein expression	Novagen
Plasmids		
pBAD33	Cm^r P_{BAD}	44
pHFAB	pBAD33/PomAB	45
pTSK37	pBAD33/PomAB (Δ41-120)	46
pTSK6	pET-22b(+)-pomAB-his	S. Kojima
pCold4	Amp^r P_{cspA} (Cold shock expression vector)	TaKaRa
pCold4-pomAB-His6	pCold4/PomAPomB-his6	This study

Amp^r, ampicillin resistant; Rif^r, rifampin resistant; Cm^r, chloramphenicol resistant; Pof^+, normal polar flagellar formation, Pof^-, defective in polar flagellar formation; Laf^-, defective in lateral flagellar formation; P_{BAD}, arabinose promoter; P_{espA}, *cspA* promoter.

Table S3. Hydrophobic thickness (Å)[a] of the TM helices in PomA and PomB

TM#	Subunit#/ residue pair	Initial model				Structure after 105ns MD			
		1	2[b]	3	4	1	2[b]	3	4
TM-A1	L3-G23	29.9	30.4	29.9	30.2	29.6	30.2	29.2	29.7
TM-A2	S34-Q54	29.5	29.2	30.4	29.2	32.4	29.6	29.7	30.5
TM-A3	V149-M169	32.1	33.5	31.8	31.4	28.5	31.3	28.8	33.3
TM-A4	M179-I200	33.9	34.9	34.8	34.8	28.5	37.1	31.8	31.6
TM-B	M19-S40	35.3	35.0	#[c]	#[c]	35.8	31.0	#[c]	#[c]

[a]Measured between Cα atoms of the two atoms at the border of the membrane spanning region for each TM helix. Data on membrane-spanning regions were taken from the paper[33].

[b]Channel-1, where structure change and sodium binding occurs, is formed between subunit 2 of PomA and subunit 1 of PomB.

[c] TM-B of PomB only has two subunits, 1 and 2 and there is no value.

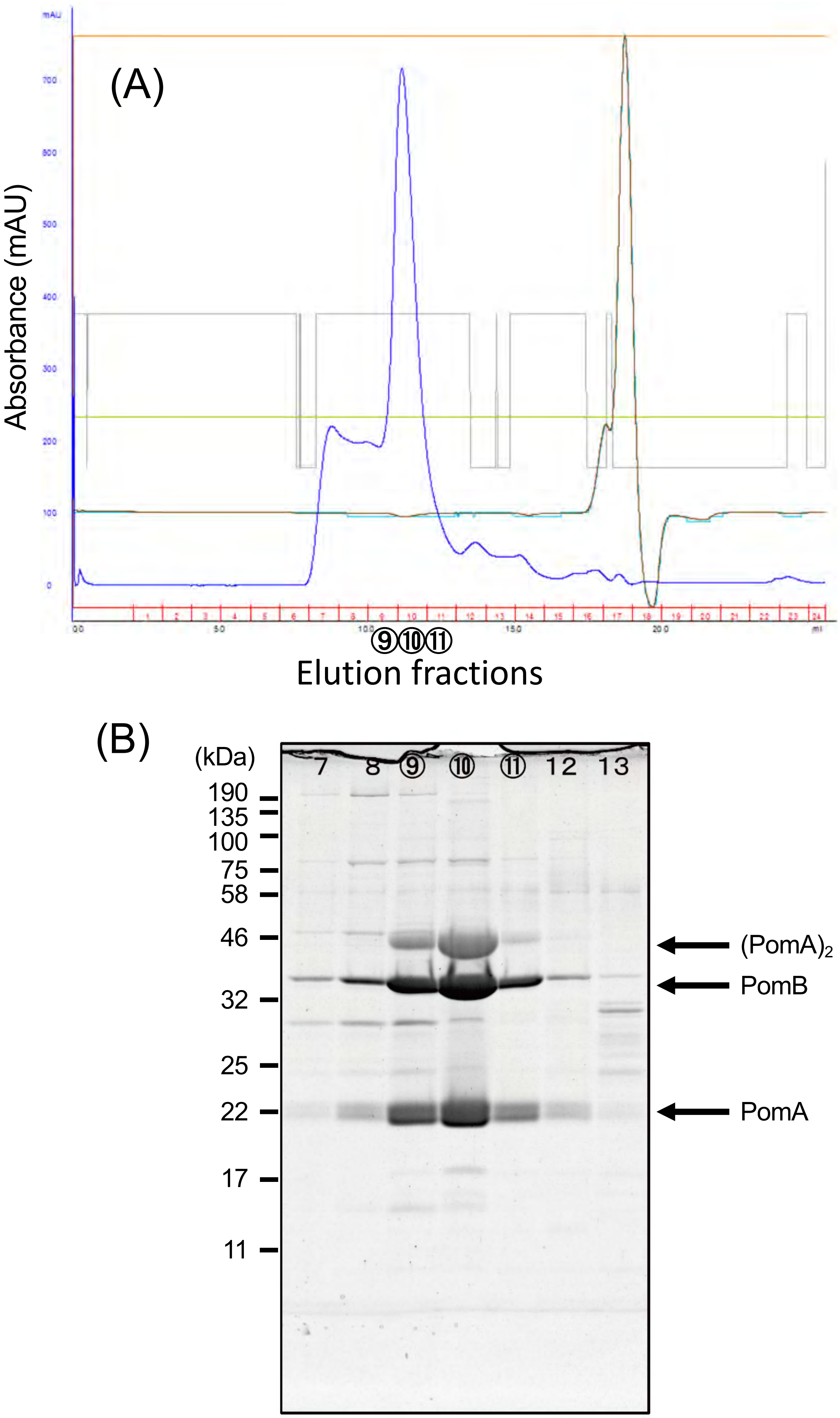

Fig. S1. (A) Elution profile of purified PomAB complex by gel filtration chromatography using Enrich SEC 650 10-300 Column (Bio-Rad). (B) The proteins of the eluted samples were separated by SDS-PAGE and detected by Coomassie brilliant blue staining.

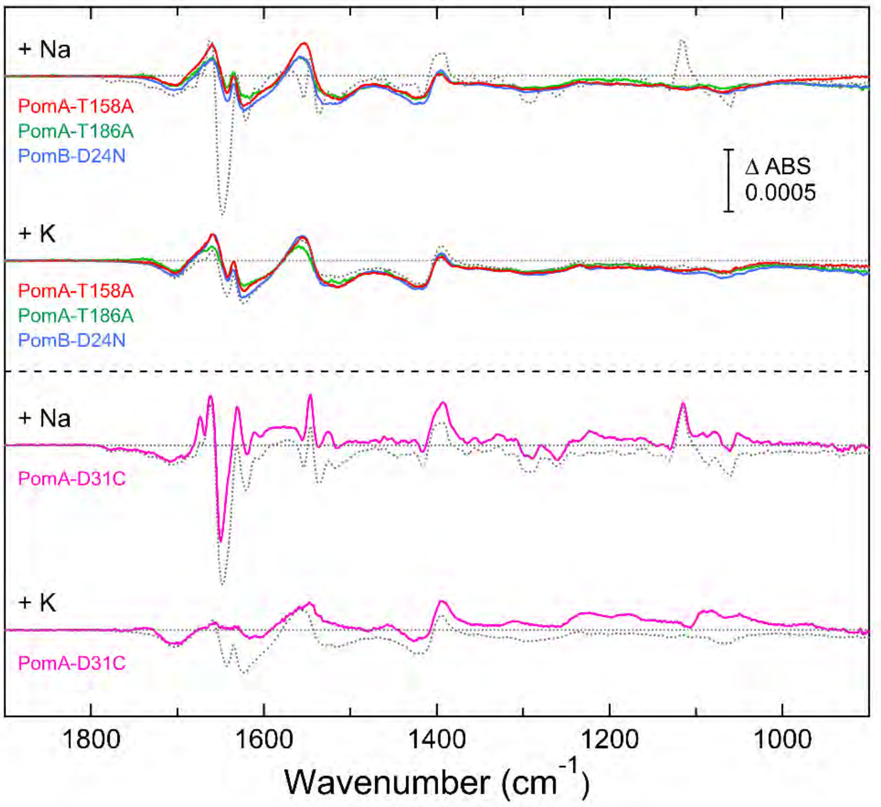

Fig. S2. Na^+- or K^+-induced difference ATR-FTIR spectra of the PomA/PomB mutants. The upper panel showed difference ATR-FTIR spectra of "20 mM -*minus*- 0 mM" NaCl (upper traces) and KCl (lower traces) were measured in the PomA-T158A (red), PomA-T186A (green) and PomB-D24N (blue) mutants. The lower panel showed the corresponding Na^+- or K^+-induced spectrum measured in PomA-D31C mutant (magenta). A perfusion buffer contained 10 mM MOPS-Tris, pH 7.0, 5 mM $MgCl_2$ in the presence/absence of 20 mM NaCl or KCl. All the spectra had been subtracted by baseline drifts as described in the experimental section. The Na^+- and K^+-induced spectra measured in the WT were overlaid for comparison (black dotted line, same as trace-a. and trace-d. in Fig. 5A).

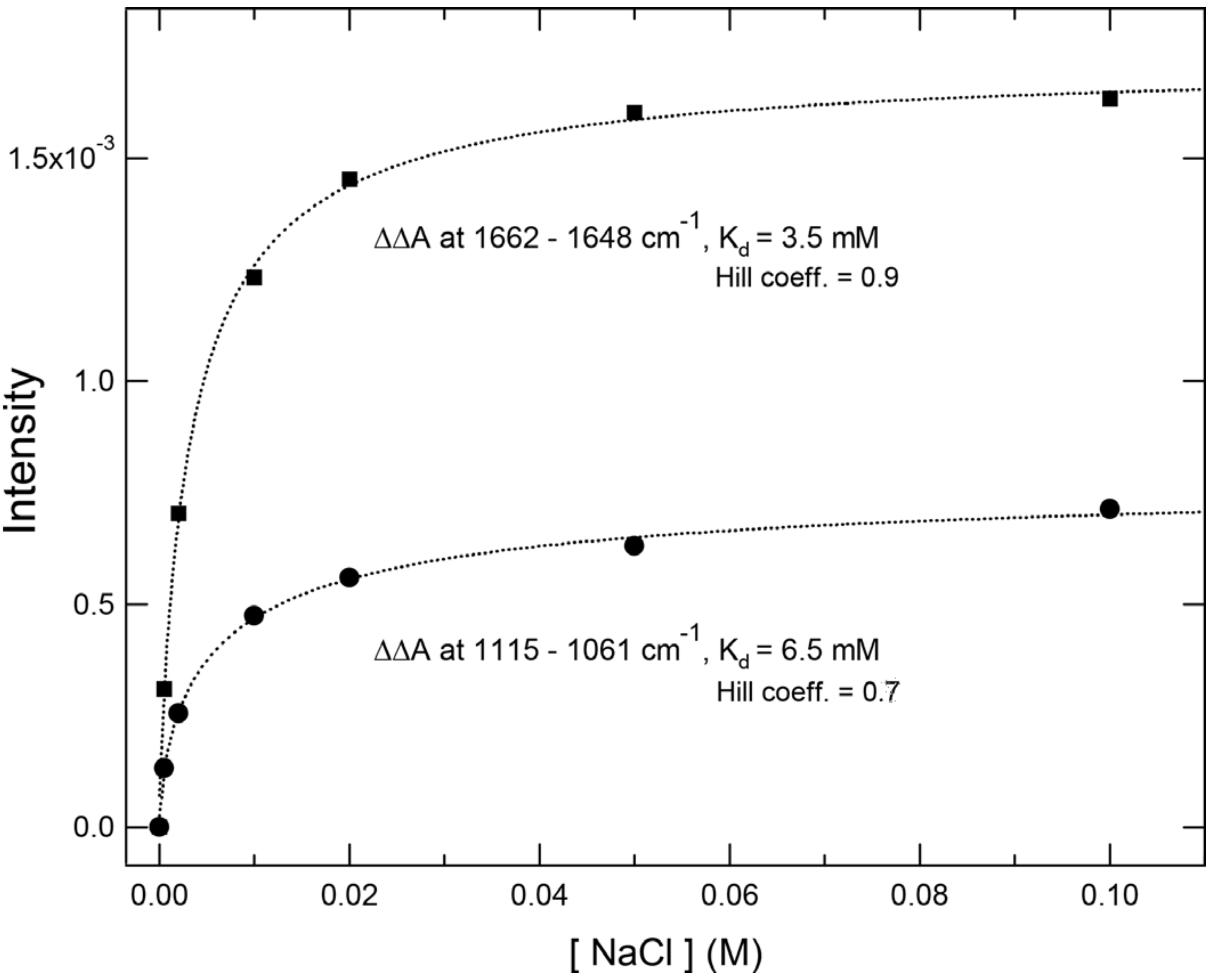

Fig. S3. Binding affinity of Na^+ to the WT PomA/PomB estimated by ATR-FTIR. The difference ATR-FTIR spectra of "Na^+-bound -*minus*- free" were measured in the WT, using the buffer (10 mM MOPS-Tris, pH 7.0, 5 mM $MgCl_2$) containing NaCl at different concentrations of 0.5, 2, 10, 20, 50, and 100 mM. After baseline subtraction, the intensities of peak/trough at 1662(+)/1648(–) cm^{-1} (square) and 1115(+)/1061(–) cm^{-1} (circle) in each spectrum were calculated and plotted against the concentration of NaCl. Both can be representative for the intensities of IR changes due to Na^+-binding to the WT, mainly in the Na^+-selective site. Dotted lines were obtained from curve fitting using the Hill equation, with parameters of dissociation constant K_d and the Hill coefficient values; 3.5 mM and 0.9 for the 1662(+)/1648(–) cm^{-1} and 6.5 mM and 0.7 for the 1115(+)/1061(–) cm^{-1}, respectively.

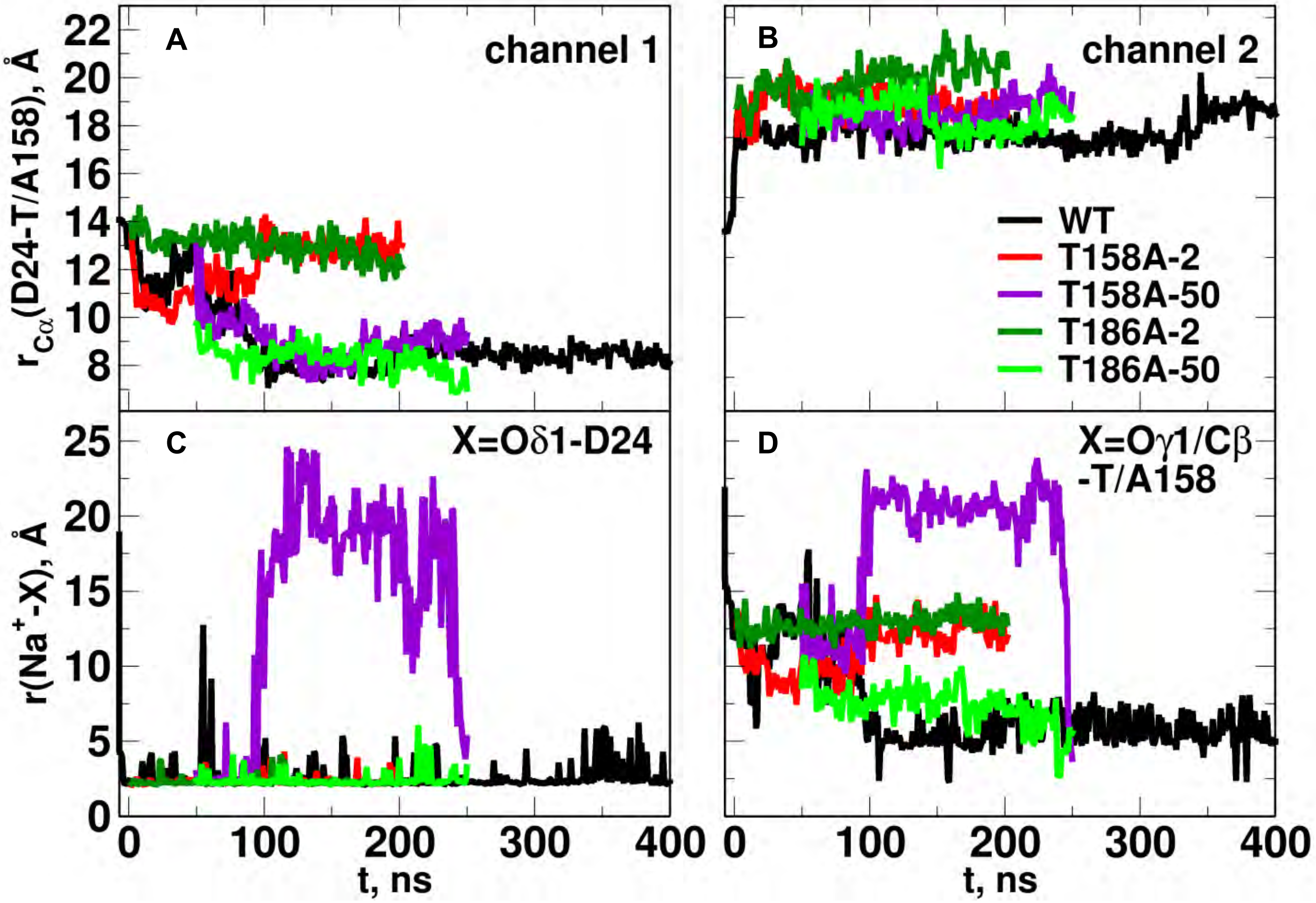

Fig. S4. (A) and (B) Distance between Cα atoms of D24 (PomB) and T/A158 (PomA) during the simulation for channel 1 and 2, respectively. Origins of mutant data are placed at the relative time in the WT simulation, from which their simulations were started (2 ns and 50 ns for T158A-2/T186A-2 and T158A-50/T186A-50, respectively). (C) and (D) Distance of D24 (Oδ1) and T/A158 (Oγ1/Cβ), respectively (in channel 1), to the nearest Na^+ atom at any time.

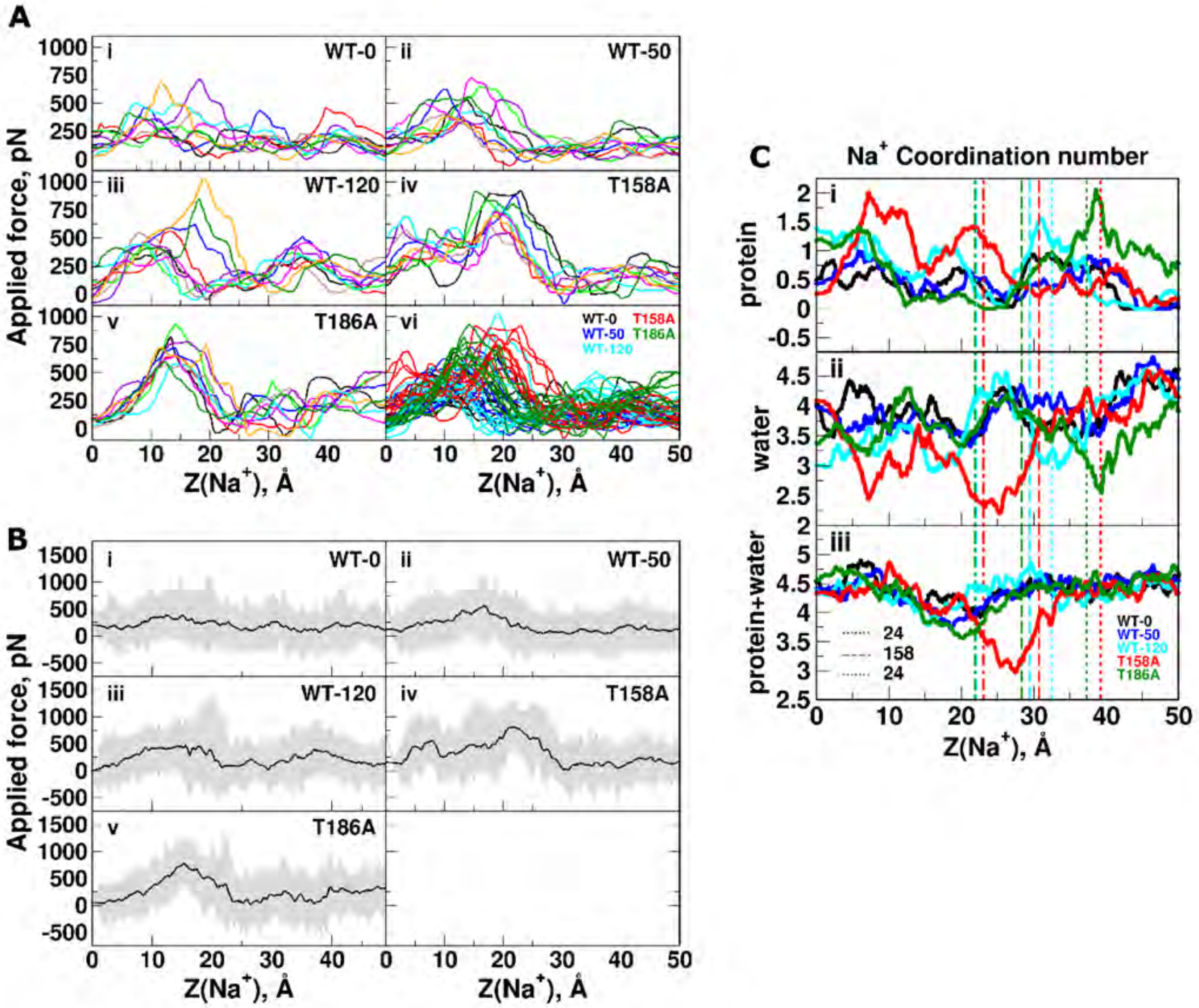

Fig. S5. (A) (i)-(v): Individual force vs. z coordinate profile for 10 SMD runs. (vi) All individual SMD profiles (plots (i)-(v)) superposed and colored according to run type, as shown in legends. (B) SMD force vs. z coordinate before averaging (grey). Data consist of superposition of data points of all 10 runs for each set. Black lines represent averaged data such that each point is an average of the 500 points around it. Total number of data points in each panel is 50110, in terms of distance, each data point is an average of points roughly 0.5Å around it. (C) Coordination number of Na^{+} ion during SMD simulation shown separately for protein atoms (i), and water molecule (ii), and combined (iii). All other details are similar to those in Fig. 7.

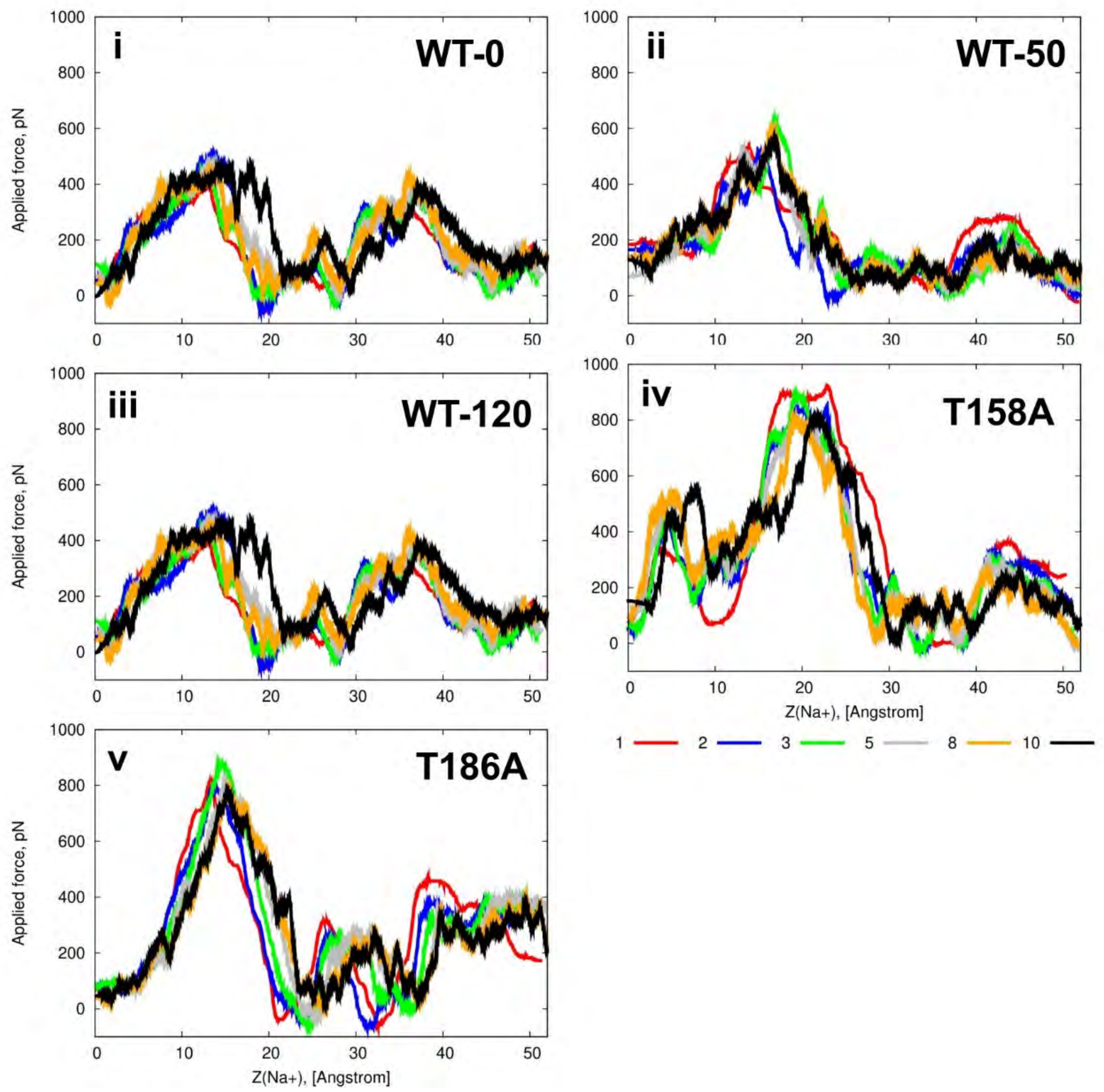

Fig. S6. Applied force during the SMD simulation vs. z coordinate of the moving Na^+ ion. For each panel, data were averaged over individual SMD simulations using the 1, 2, 3, 5, 8, and 10 first simulations. Averaging of data was performed in a similar way as in Fig. 7.

A

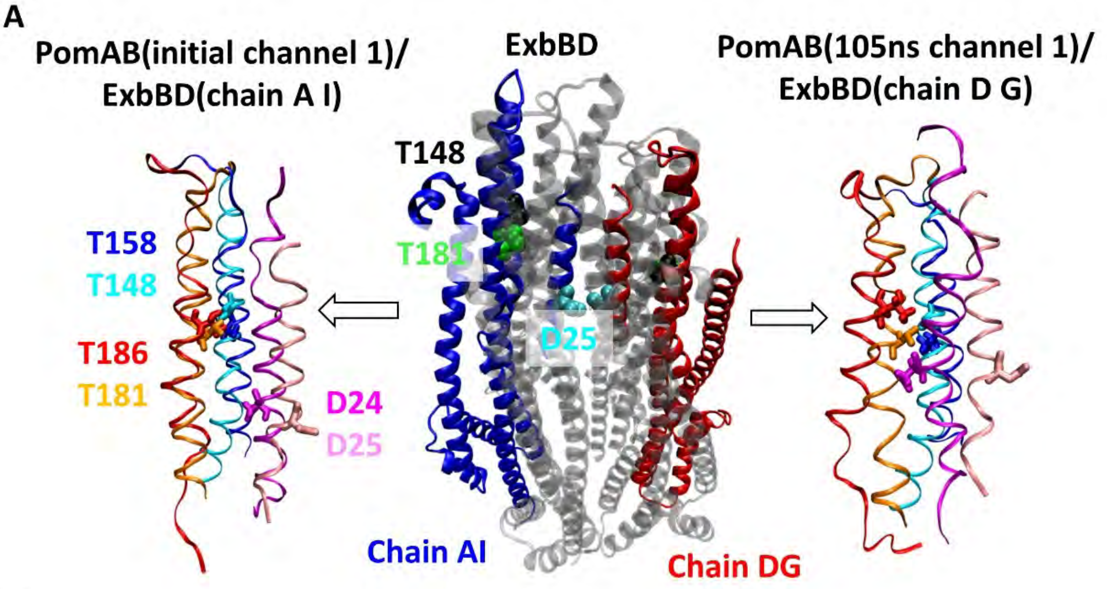

B

Sequence alignment results for PomA and ExbB

PomA	147	G	D	V	A	P	A	M	G	M	I	G	T	L	V	G	L	162
ID/SIM		G		+	+	P		+	G	+		G	T	+		G	+	
ExbB	137	G	A	I	S	P	F	V	G	L	F	G	T	V	W	G	I	152

PomA	163	V	A	M	L	-	-	-	-	-	S	N	M	D	D	P	K	173
ID/SIM		+									+				+			
ExbB	153	M	N	S	F	I	G	I	A	Q	T	Q	T	T	N	L	A	168

PomA	174	A	I	G	P	A	M	A	V	A	L	L	T	T	L	Y	G	189
ID/SIM			+		P		+	A		A	L	L		T			G	
ExbB	169	V	V	A	P	G	I	A	E	A	L	L	A	T	A	I	G	184

C

ExbBD		PomAB (105ns)	
Channel	r(T148-D25)	Channel	r(T158-D24)
CH	9.5	1	8.8
DG	14.6		
AI	20.5	2	17.7

Channel	r(T181-D25)	Channel	r(T186-D24)
CH	9.9	1	8.5
DG	13.3		
AI	17.2	2	21.2

Fig. S7. (A) Left and right: channel regions of PomAB (blue, red, and magenta) and ExbBD (cyan, orange, and pink) [36] fitted according to channel 1 of PomA (TM3) with the TM regions (residues 129-196) of ExbBD (chain A, left and chain D, right). PomAB structures used for fitting are the initial model (left) and an MD snapshot after 105ns of simulation (right). Thr and Asp residues of both proteins are presented in stick representation and color-coded as shown in the left panel. Each helix is colored with the same color as the emphasized residues on it. Middle: ExbBD hexamer (PDB id: 5ZFU) emphasizing chains A and I (blue) and chains D and G (red). Thr and Asp residues are presented as spheres and color-coded as shown in the figure. Channels were assigned as follows. Each ExbD unit was paired with the three ExbB units closest to it. An alignment was performed against PomAB channel 1 for all possibilities. The ExbB unit that resulted in the best fit (lowest RMSD) was selected as the matching pair for each ExbD unit. (B) Sequence alignment results for PomA and ExbB. Alignment performed with the protein-protein BLAST® (blastp) using the Compositional matrix adjustment method. %identity was determined as 27% (13/48), %similarity was 50% (24/48) 5 gaps (10%) were inserted. The total BLAST score was 20.8. Range used for alignment in PomA were residues 144-205 (TM3) and for ExbB, residues 129-196 (TM part of the channel). Information about identities and similarities is shown in the ID/SIM rows. A letter indicates the 1-letter code of an identical residue, a plus sign indicates similar residues. (C) Distances (between Cα atoms, in Angstrom) between Thr and Asp residues in PomAB and ExbBD channels. Coordinates for ExbBD were taken from the Cryo-EM hexameric structure (PDB id: 5ZFU), coordinates for PomAB taken from a snapshot of the WT-MD simulation after 105ns. T148 and T181 belong to ExbB, D25 belongs to ExbD, T158 and T186 belong to PomA, and D24 belongs to PomB. Channels were assigned as described in (A).

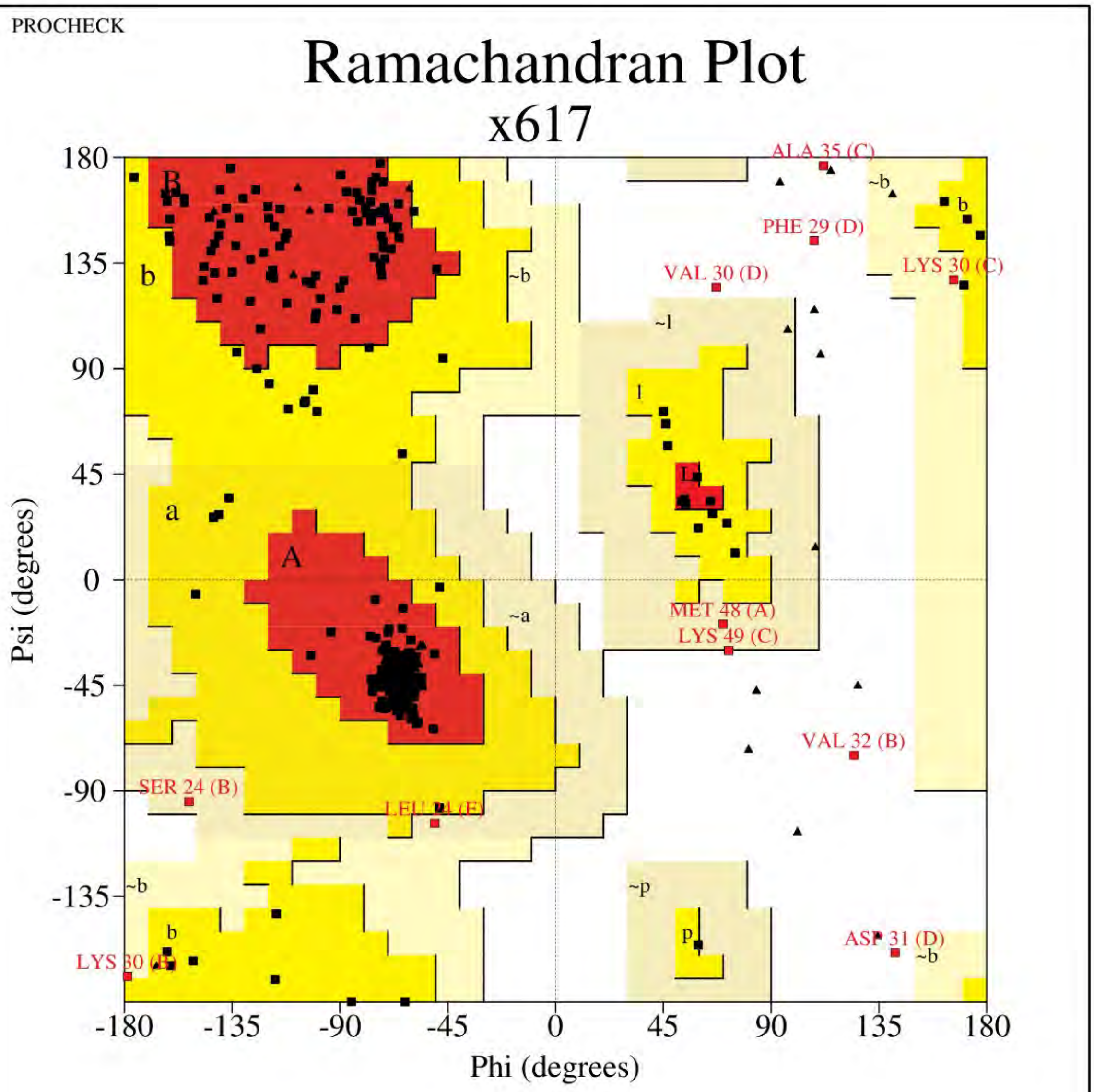

Plot statistics

Residues in most favoured regions [A,B,L]	388	88.6%
Residues in additional allowed regions [a,b,l,p]	39	8.9%
Residues in generously allowed regions [~a,~b,~l,~p]	5	1.1%
Residues in disallowed regions	6	1.4%
	----	------
Number of non-glycine and non-proline residues	438	100.0%
Number of end-residues (excl. Gly and Pro)	18	
Number of glycine residues (shown as triangles)	68	
Number of proline residues	22	

Total number of residues	546	

Based on an analysis of 118 structures of resolution of at least 2.0 Angstroms and R-factor no greater than 20%, a good quality model would be expected to have over 90% in the most favoured regions.

x617_01.ps

Fig. S8. Ramachandran plot for the final model of PomAB used for the MD simulations. Analysis was performed in Procheck [54]. Due to renumbering of residues some residues are presented in the plot with different residue numbers as follows. PomA-S167 presented as S24, PomA-K173 as K30, PomA-A178 as A35, and PomB-L35 as L24. Letters in parenthesis indicate the chain number, chains A-D for PomA and chains E, F for PomB.

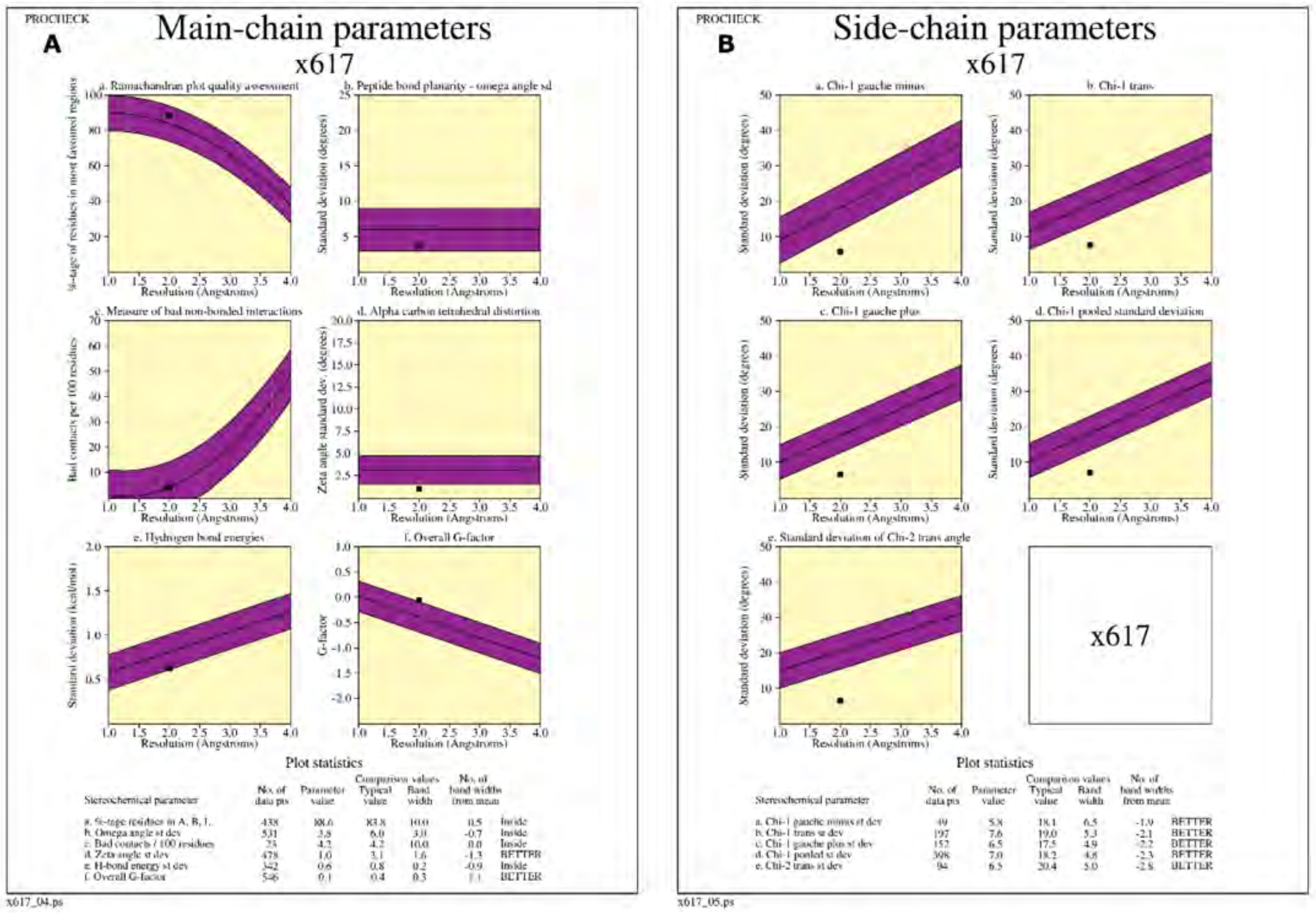

Fig. S9. Main-chain (A) and side-chain (B) parameters for the final model of PomAB used for the MD simulations. Analysis was performed in Procheck [33]. The value for the target structure is represented as a solid black square. Purple band represents the results from well-defined structures at each resolution. The width of the band corresponds to a variation of one standard deviation about the mean.

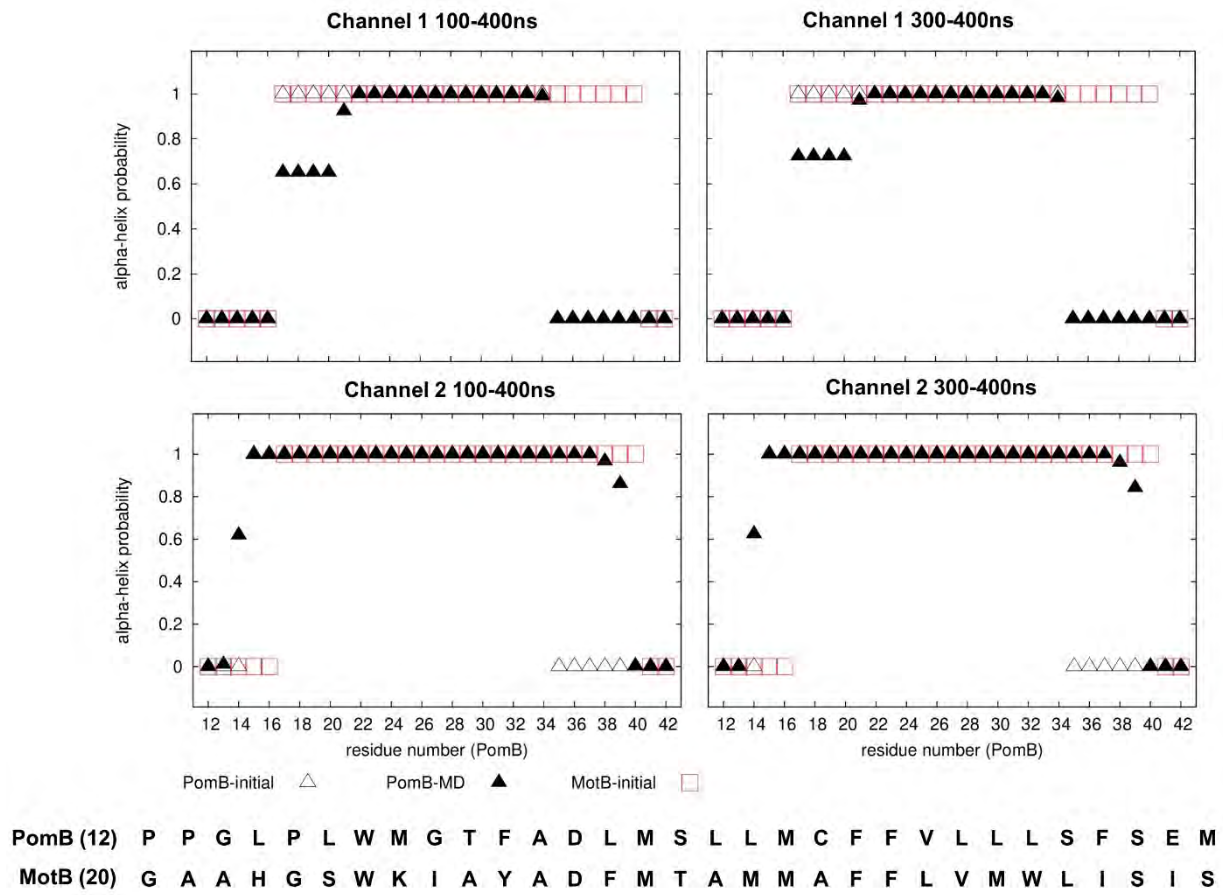

Fig. S10. Helix propensities of TM-B residues of PomB during MD simulation, for the initial (modeled) structure of TM-B of PomB, and for the initial structure of TM-B of MotB (after the second stage of "Evaluation & selection" in Fig. S1 in the literature[19]). Residue numbers are shown for PomB sequence, sequence alignment with MotB for the relevant parts are shown in the bottom. Data shown for channels 1 and 2 at the top and bottom panels, respectively, and for 100-400ns and 300-400ns on the right and left panels, respectively. For residues in which the propensities in the initial state and during the simulation are equivalent, the "PomB-initial" marker overlaps the "PomB-MD" marker and is not visible.

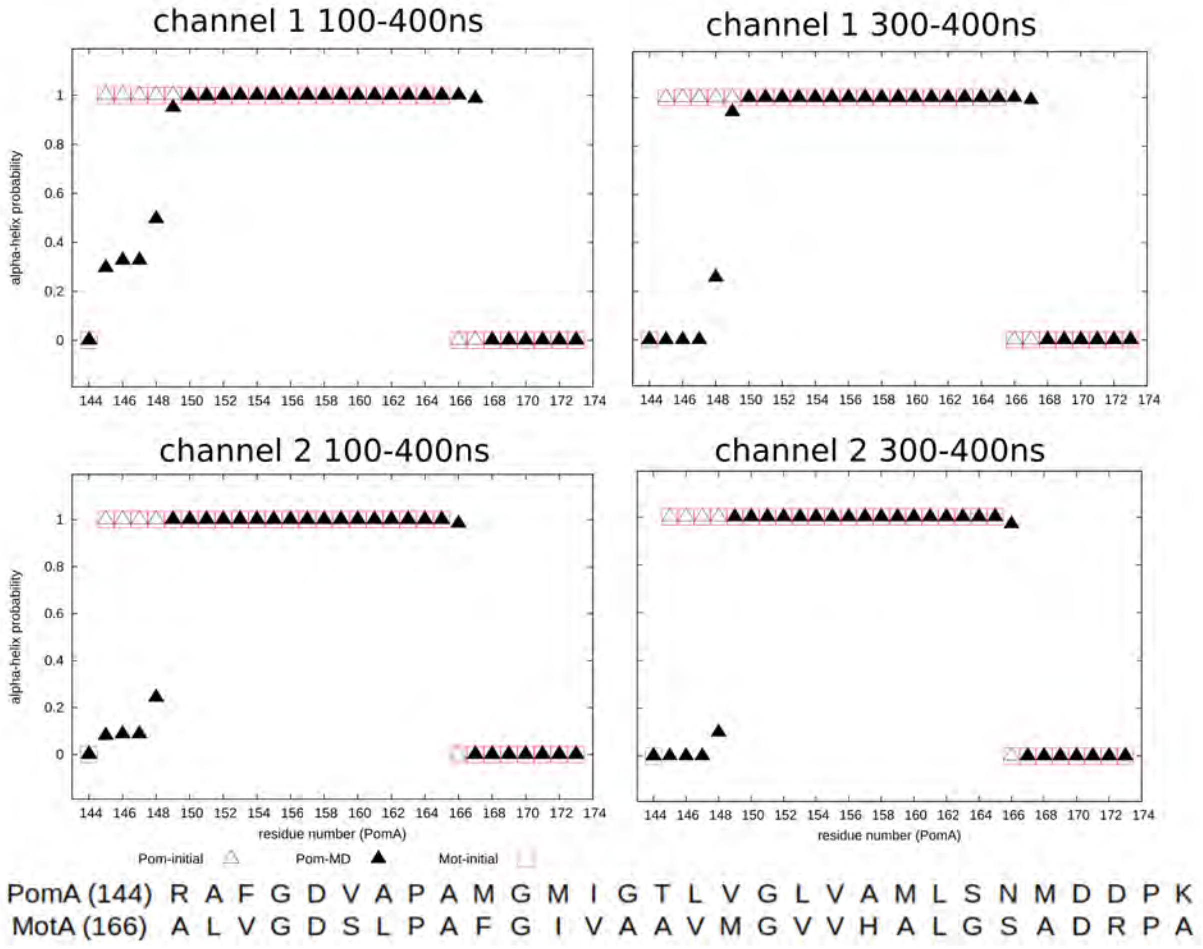

Fig. S11. Helix propensities of TM-3 residues of PomB during MD simulation, for the initial (modeled) structure of TM-3 of PomB, and for the initial structure of TM-3 of MotB (after the second stage of "Evaluation & selection" in Fig. S1 in the literature[19]). Other descriptors are the same as for Figure S10.

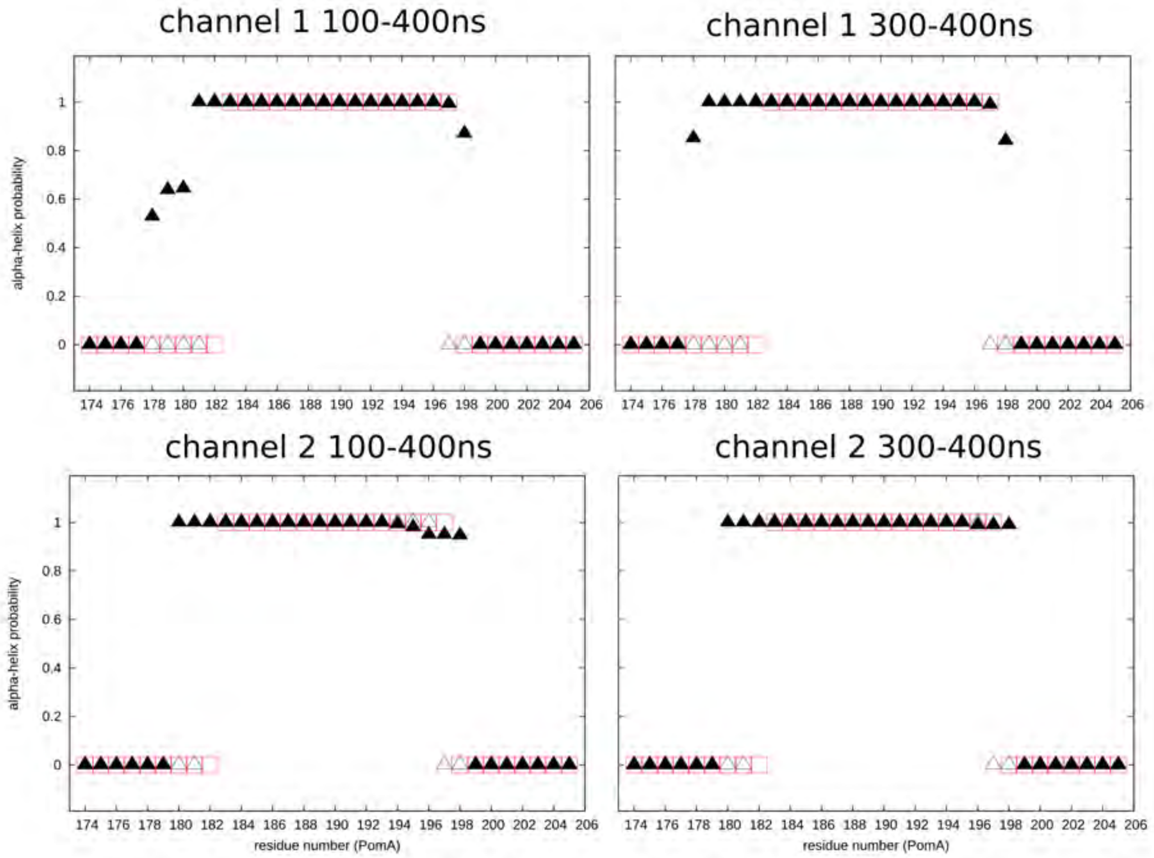

PomA (174) A I G P A M A V A L L T T L Y G A I L S N M V F F P I A D K L S
MotA (197) E L G A L I A H A M V G T F L G I L L A Y G F I S P L A S V L R

Fig. S12. Helix propensities of TM-4 residues of PomB during MD simulation, for the initial (modeled) structure of TM-4 of PomB, and for the initial structure of TM-4 of MotB (after the second stage of “Evaluation & selection” in Fig. S1 in the literature[19]). Other descriptors are the same as for Figure S10.

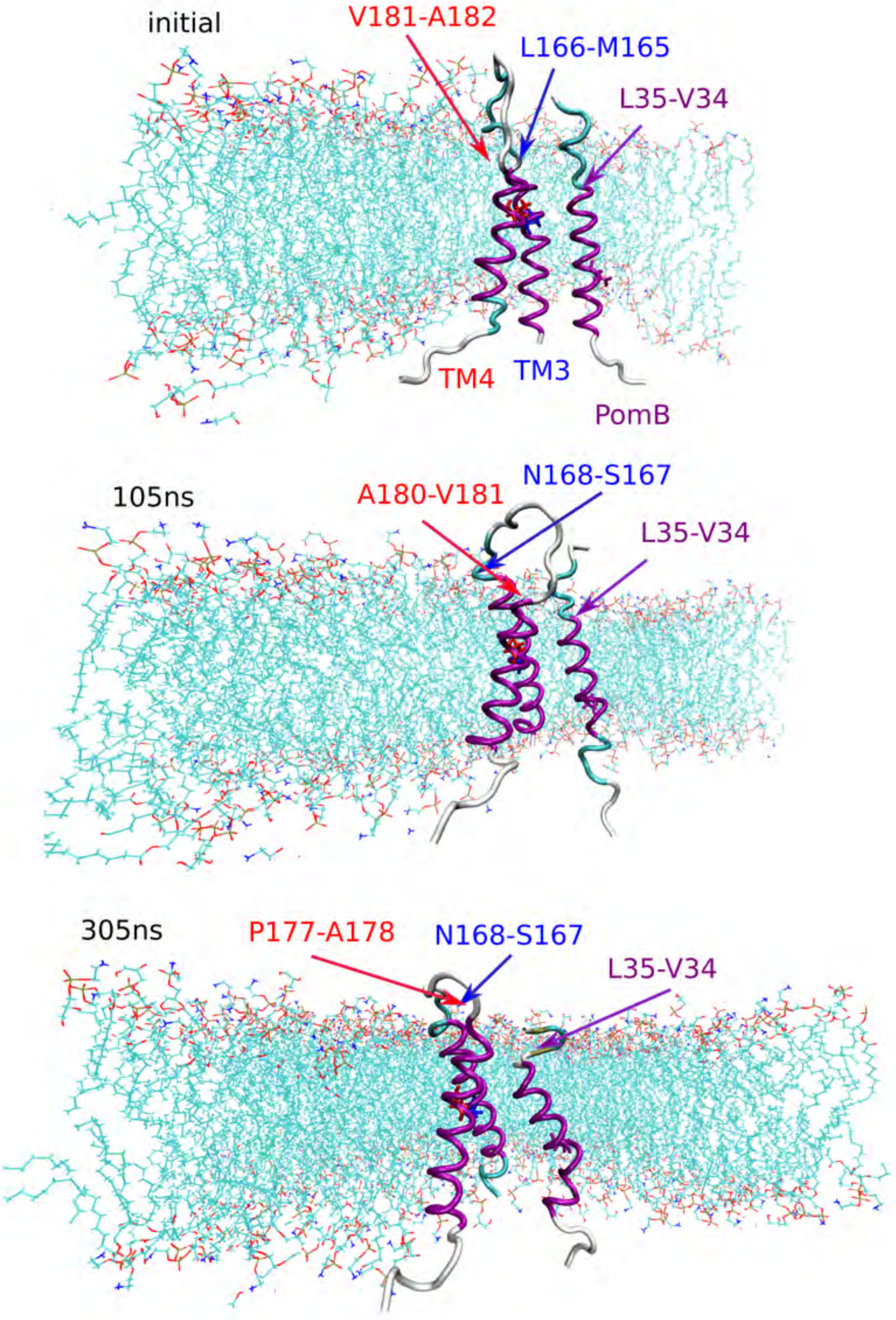

Fig S13. TM-3 and TM-4 of PomA and TM-B of PomB with the embedding membrane (for clarity of the figure, the front region of the membrane is not shown) for channel 1, shown for the initial (modeled) structure (top) and for snapshots from the MD simulation after 105 ns (middle) and 305 ns (bottom). Protein regions are colored according to secondary structure (alpha helix in purple, turn in cyan and coil in white). T158 of TM-3, T186 of TM-4 and TM-B of PomB are shown in stick representation in blue, red and purple, respectively. Arrows mark the border between helical (bottom) and non-helical regions. Residue pairs between which helices are broken written above the arrows where the residue name on the left is the top residue (the non-helical residue).

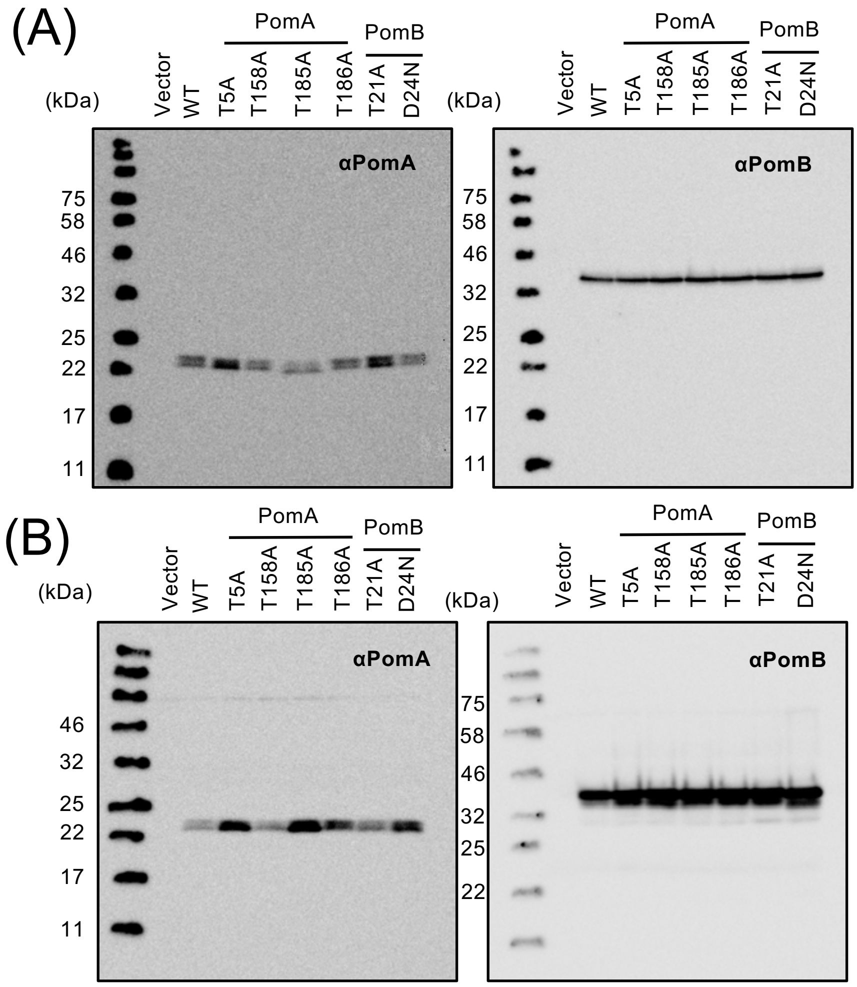

Fig. S14. Full length blots of cropped images in the main text. A part of the blots of (A) and (B) are shown in Fig. 2D and Fig. 3B, respectively. Marker, molecular weight marker (unit: kDa).

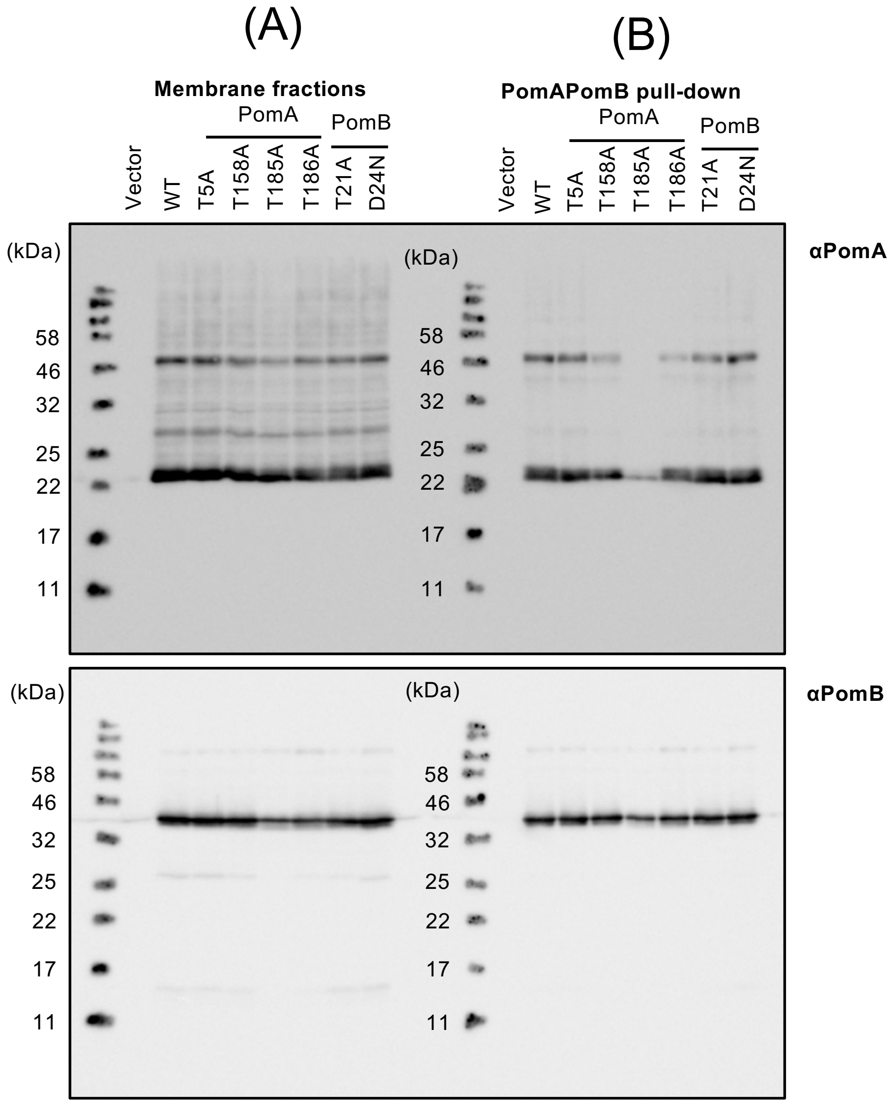

Fig. S15. Full length blots of cropped images in the main text. A part of the blots of (A) and (B) are shown in Fig. 4A and B, respectively. Marker, molecular weight marker (unit: kDa).
